# Socioexposomics of COVID-19 across New Jersey: a comparison of geostatistical and machine learning approaches

**DOI:** 10.1038/s41370-023-00518-0

**Published:** 2023-02-01

**Authors:** Xiang Ren, Zhongyuan Mi, Panos G. Georgopoulos

**Affiliations:** 1 Environmental and Occupational Health Sciences Institute (EOHSI), Rutgers University, Piscataway, NJ 08854 USA; 2 Department of Chemical and Biochemical Engineering, Rutgers University, Piscataway, NJ 08854 USA; 3 Department of Environmental and Occupational Health and Justice, Rutgers School of Public Health, Piscataway, NJ 08854 USA; 4 Department of Environmental Sciences, Rutgers University, New Brunswick, NJ 08901 USA

**Keywords:** COVID-19, Social/environmental health disparities, Exposome and socioexposome, Explainable machine learning, Bayesian geospatial modeling

## Abstract

**Background:**

Disparities in adverse COVID-19 health outcomes have been associated with multiple social and environmental stressors. However, research is needed to evaluate the consistency and efficiency of methods for studying these associations at local scales.

**Objective:**

To assess socioexposomic associations with COVID-19 outcomes across New Jersey and evaluate consistency of findings from multiple modeling approaches.

**Methods:**

We retrieved data for COVID-19 cases and deaths for the 565 municipalities of New Jersey up to the end of the first phase of the pandemic, and calculated mortality rates with and without long-term-care (LTC) facility deaths. We considered 84 spatially heterogeneous environmental, demographic and socioeconomic factors from publicly available databases, including air pollution, proximity to industrial sites/facilities, transportation-related noise, occupation and commuting, neighborhood and housing characteristics, age structure, racial/ethnic composition, poverty, etc. Six geostatistical models (Poisson/Negative-Binomial regression, Poison/Negative-Binomial mixed effect model, Poisson/Negative-Binomial Bersag-York-Mollie spatial model) and two Machine Learning (ML) methods (Random Forest, Extreme Gradient Boosting) were implemented to assess association patterns. The Shapley effects plot was established for explainable ML and change of support validation was introduced to compare performances of different approaches.

**Results:**

We found robust positive associations of COVID-19 mortality with historic exposures to NO_2_, population density, percentage of minority and below high school education, and other social and environmental factors. Exclusion of LTC deaths does not significantly affect correlations for most factors but findings can be substantially influenced by model structures and assumptions. The best performing geostatistical models involved flexible structures representing data variations. ML methods captured association patterns consistent with the best performing geostatistical models, and furthermore detected consistent nonlinear associations not captured by geostatistical models.

**Significance:**

The findings of this work improve the understanding of how social and environmental disparities impacted COVID-19 outcomes across New Jersey.

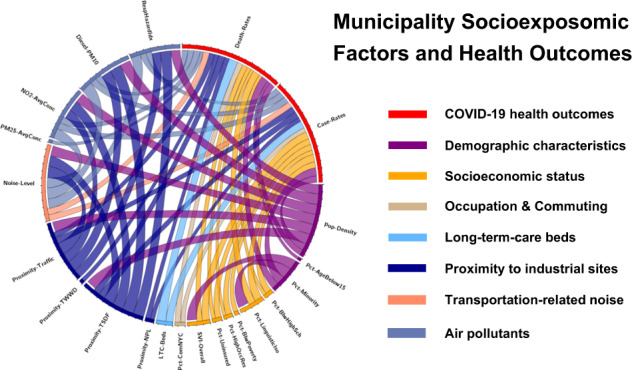

## Introduction

COVID-19 had caused over 6.7 million deaths worldwide as of the end of 2022 (https://covid19.who.int/) and it is expected to have long-lasting impacts on global health [1]. Various ecological and individual-level studies have been conducted [2–7], exploring associations of COVID-19 morbidity and mortality with environmental and social determinants of health. Most studies focused on a single stressor or a single group of stressors; an exception is an external exposome-wide association study of county-level COVID-19 mortality across the contiguous US [8]. However, it is recognized that it is important to establish integrated frameworks that simultaneously consider heterogeneous stressors at multiple scales [9, 10], in order to assess risk factors associated with health disparities.

It has also been recognized that COVID-19 is not just a pandemic but a syndemic, involving interactions of multiple factors and conditions [11]; advanced data-driven approaches are therefore needed to capture complex underlying association patterns. The socioexposome provides a multidisciplinary framework for accomplishing this by integrating the concepts of the exposome and precision health with socioeconomic and behavioral factors to better understand repercussions of regulatory and corporate practices on public health and social justice [12]. Within the exposome [13], the socioexposome specifically focuses on external exposures and socioeconomic conditions and the need for community engagement in exposome science [14]. This framework is particularly relevant to modeling spatial heterogeneities of COVID-19, that are driven by a multitude of correlated and interacting biological, demographic, socioeconomic and environmental factors [15]. For instance, racial/ethnic minorities are more likely to live near industrial sites and pollution hotspots, and these communities are therefore exposed to higher levels of pollutants and other stressors that may exacerbate the severity of COVID-19 [16, 17]; built environment factors such as green space and noise exposure were also found to be related to disparities in COVID-19 outcomes [18, 19].

Comparisons of linear statistical models have been conducted in exposome-health studies [20–22], showing that no method is consistently superior to others across different data sets and criteria: selection of efficient models should be based on specific tasks and data behaviors [23]. Various models have been used to quantify associations between stressors and COVID-19 outcomes. Ordinary linear regression (OLR) was applied to model numbers of cases/deaths [24, 25], but inappropriate Gaussian assumptions may lead to overoptimistic *p*-values and erroneous estimates of association [26]. To alleviate this issue, generalized linear regression (GLR), such as Poisson regression and Negative-Binomial (NB) regression, can be used [2, 27]. However, GLR may still not be sufficient for describing health outcomes with high variabilities due to overdispersion, group randomness, spatial autocorrelation, etc. Failure to consider these elements may drastically affect interval estimates of different variables. It is therefore needed to systematically evaluate and compare the performance of geospatial models in assessing socioexposomic patterns of COVID-19.

Machine Learning (ML) has been gaining popularity in environmental health sciences [28]. However, most ML applications are pursued for prediction rather than for association inference [29]. Barrera-Gómez et al. [21] compared seven algorithms with respect to detecting interactions in exposome-health associations, and found boosted regression trees to have the lowest predicted *R*^2^ compared to other linear framework algorithms. Boosted regression was reported to have undesirably higher false positive discoveries for variable selection than other linear models in a simulated case-control study [30]. Since these results were generated with analyses assuming linear data structures, they do not truly reflect the strengths of ML. Supervised ML can capture underlying nonlinear associations and interactions using flexible model architectures and efficient algorithms [31]. ML has been considered less interpretable than linear models, a concern that has limited its application in association studies. Recently, metrics such as variable importance were introduced to compare performance and consistency of different ML algorithms in environmental health association studies [32, 33]. However, important questions remain regarding how these algorithms perform with respect to identifying and quantifying complex patterns of socioexposome-health associations.

**Fig. 1:**
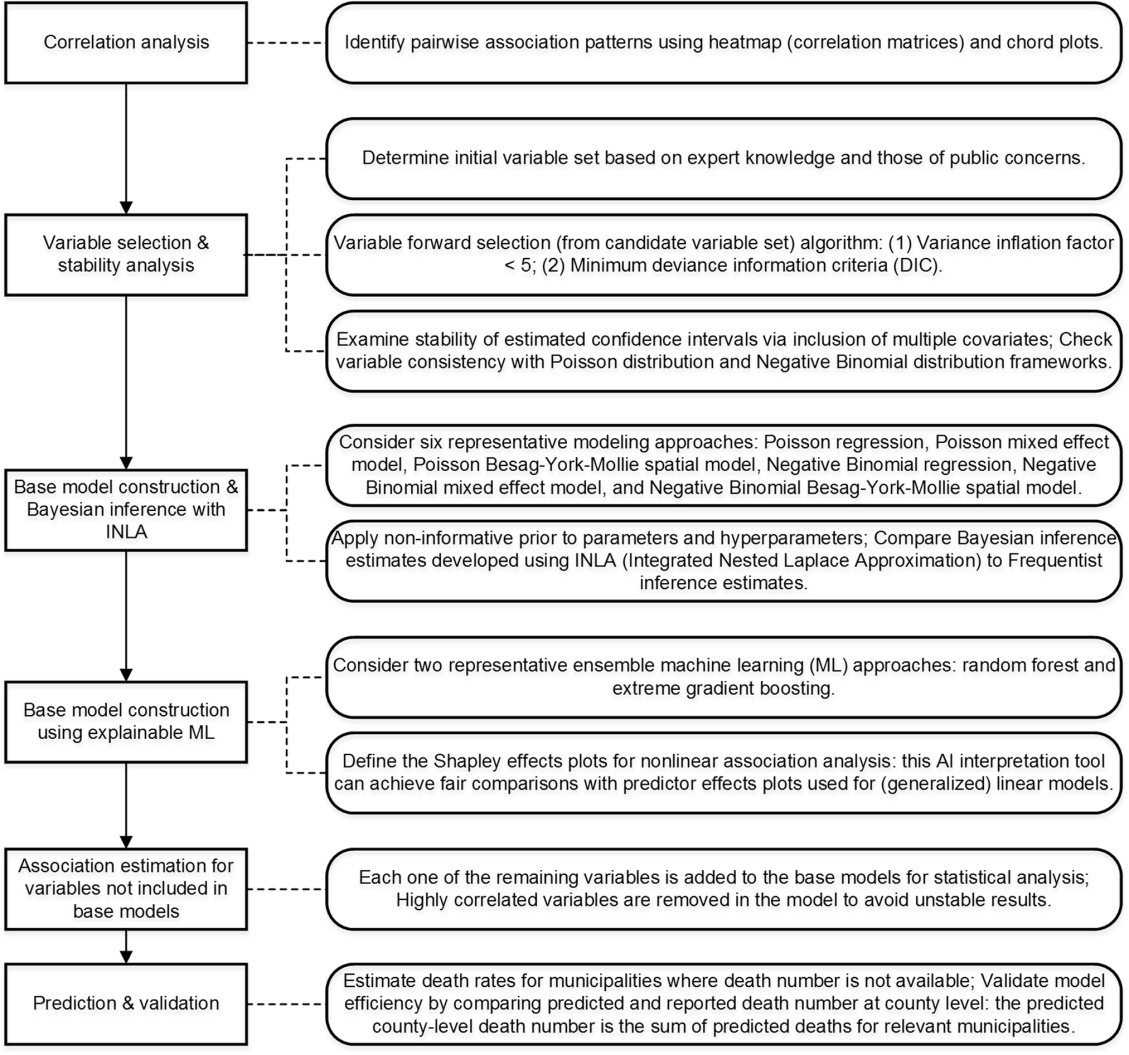
Flow diagram of the COVID-19 socioexposomic modeling framework. Six main components/procedures are shown in the left rectangular blocks, with descriptions of each component given on the right.

The present study implemented a unified framework (Fig. 1) applying and comparing eight representative ML and geostatistical models with structure hierarchy, for health impact assessment and prediction of COVID-19 adverse health outcomes at municipality level across New Jersey.

## Materials and methods

### Study settings

The spatial domain of this study covered the 565 municipalities of the State of New Jersey (Fig. S1). We focused on the first wave of the pandemic (March to September 2020) to exclude effects from factors such as vaccination and virus variants. It should be noted that New Jersey was one of the earliest and largest hotspots for COVID-19 (Fig. S2), with the highest per capita fatalities from the first wave of COVID-19. New Jersey is one of the most ethnically, socioeconomically and environmentally diverse States; while it has on average the highest population density in the nation, its 565 municipalities include urban centers, suburban sprawl, shore towns, an aging industrial infrastructure, as well as agricultural land and forested areas. So, New Jersey constitutes a remarkably heterogeneous environmental landscape that has often been considered a “microcosm” representative of conditions occurring across the entire contiguous US [34].

### Data sources

The variables and data sources used in this study are summarized in Table S1.

#### COVID-19 cases/deaths

Cumulative COVID-19 cases/deaths as of September 24, 2020 were available from local health departments across New Jersey. Deaths in long-term-care facilities (LTCF) were provided by NJDOH; see Text S1 for calculation of mortality rates with/without LTCF deaths.

#### Demographic characteristics

Demographic factors were retrieved from the 2015–2019 US Census Bureau American Community Survey (ACS). These factors include population density, age groups, racial and ethnic communities, etc.

#### Socioeconomic status

Socioeconomic factors considered here included education, language isolation, household crowding, poverty, disability, unemployment, uninsured community, social vulnerability index (SVI), etc. Please refer to Text S1 for details on individual socioeconomic variables and combined indices.

#### Air pollutants

Metrics for three criteria air pollutants were considered in this study: Annual average PM_2.5_ and summer seasonal average of daily maximum 8-hour ozone concentrations (2016) were retrieved from the EJSCREEN at the block group level. Annual averages of daily maximum 1-h NO_2_ concentrations (2016) were made available by Di et al. [35] at 1 × 1 km^2^ resolution. We also considered twenty air toxics and relevant risk indices retrieved from the USEPA NATA estimates; see Text S1 for details.

#### Proximity to industrial sites

Proximity data associated with significant industrial sites and facilities (2019) were extracted from EJSCREEN. The spatial distribution of power plants in 2020 was available from NJDEP; inverse distance weighting by facility size was used to calculate proximity to energy generating units.

#### Transportation-related noise

Noise estimates from the transportation sector, i.e., aviation, roadway, and passenger rail, were made available by the Bureau of Transportation Statistics providing 24-h equivalent A-weighted noise levels for 2018 at 30 × 30 m^2^ resolution averaged at the municipality level.

#### Occupation and commuting

Occupation data were obtained from the Longitudinal Employer-Household Dynamics database for 2018. Occupation types considered include health care, food service, transportation, retail and wholesale. Commuting data to residents’ workplaces (either in another county or in New York City) were also extracted from LEHD.

#### Other

The number of licensed long-term-care beds for each facility (2020) was acquired from the NJDOH, and summed up for each municipality. Data from the Agency for Healthcare Research and Quality were used to calculate numbers of full-service restaurants and supermarkets per 1000 residents.

### Statistical/machine learning approaches

#### Geostatistical models with structure hierarchy

We used six statistical/geospatial models (Table S2) with flexible random components: Poisson regression, Poison mixed effect model, Poisson Bersag-York-Mollie (BYM) spatial model, Negative-Binomial (NB) regression, NB mixed effect model, NB BYM spatial model. Poisson regression assumes the response to be Poisson distributed, where the log mean is modeled as a linear combination of covariates (linear predictor). The Poisson distribution has equal mean and variance, limiting the model’s ability to capture high variability (overdispersion) in count data. NB regression imposes a hierarchical structure on the Poisson mean and includes a dispersion parameter to capture greater variability. Both methods link the fixed effect (linear predictor) to the response, ignoring potential differences between samples or sample groups. Poisson/NB mixed effect models simultaneously consider fixed and random effects to better simulate data behaviors; herein, a random intercept varied by five geographic regions within New Jersey (Fig. S1) was included.

Since the ordinary random effect component cannot sufficiently capture spatial patterns, spatial mixed Poisson/NB regression, incorporating both random and spatial components, was used to capture spatial heterogeneities. We employed a municipality-specific ordinary random effect component to describe non-spatial heterogeneity and the intrinsic conditional autoregressive structure to formulate the spatial component (a.k.a., Poisson/NB BYM spatial models). We constructed a 565 × 565 adjacency matrix $\left( {w_{i,j}} \right)_{565 \times 565}$ based on neighbor relationships to characterize spatial correlations, i.e., *w*_*i*,*j*_=1 if municipality *i* is adjacent to municipality *j* (*j* ≠ *i*) and *w*_*i*,*j*_ = 0 otherwise. Spatial correlations are estimated using the 356 municipalities with available death data, while predictions for the remaining 209 municipalities are generated from estimated spatial correlations. Frequentist and Bayesian inference (Text S2) were applied to fit these models. In the Bayesian models, non-informative priors were introduced, using default values specified in R-INLA (https://www.r-inla.org/).

#### Ensemble machine learning models

Two widely used machine learning (ML) models, i.e., Random Forest (RF) and Extreme Gradient Boosting (XGBOOST) were also implemented in this study. The models utilize two ensemble algorithms (bagging and boosting) for improved performance. RF averages outputs from multiple independent trees, where each tree grows with a subsample set from bootstrap resampling and partitions data using the optimal features in a random subset: aggregation of these less correlated trees can significantly increase model stability and accuracy. XGBOOST combines the scaled outputs from multiple successive trees, where each tree grows based on residuals (i.e., gradient of loss) from previous trees and “boosts” via gradient descent; the outputs scaled with the learning rate can promote establishment of more complementary trees to reduce model bias and avoid overfitting.

These two ensemble ML models involve flexible and scalable components/modules (e.g., regularization term used by LASSO, tree pruning used by decision tree, parallel scheme for big data) to further enhance model accuracy/robustness, and thus generally perform better than other ML methods in regression [23]. It should be noted that ML requires determining multiple hyperparameters to truly utilize the “learning capability” of the algorithms. Herein, we used repeated coarse-fine grid search for hyperparameter tuning to maximize model performance. The best (parsimonious) structures were selected based on the minimum predicted *R*^2^ for a fivefold cross validation set. The tuned hyperparameters selected for the two models are provided in Table S3.

### Variable selection

A forward stepwise algorithm starting with a predefined set was used for variable selection. This algorithm was implemented in two frameworks (Poisson and NB regression). To prevent collinearity and to ensure stable estimates, in each step, variables with variance inflation factor below 5 were considered and selected according to the deviance information criterion (DIC) [36]. The two regression frameworks obtained consistent results with ten selected variables: % population (age > 64), % minority, % below high school education, median gross rent, population density, % occupation (high risk), PM_2.5_ average concentration, ozone seasonal DM8HA, % high occupancy residence, and % unemployed. Base models were constructed with these variables; each of the remaining variables was then added to the base models for statistical analysis. For fair comparison, the ten variables were also used to build the ML base models.

### Association interpretation and quantification

Linear regression and its extensions use simple structures to ensure straightforward interpretation in effect analysis. The Poisson/NB modeling frameworks assume a log linear structure: ${{{{{{{\mathrm{log}}}}}}}}(y) = \beta _0 + \beta _1x_1 + \ldots + \beta _ix_i + \ldots + \beta _px_p$, where *β*_*i*_ denotes the regression coefficient of the *i*th variable *x*_*i*_. Based on this structure, it can be stated that a unit increase in *x*_*i*_ is associated with 100[exp(*β*_*i*_) − 1]% increase in response *y*. The predictor effects plot can be used to provide graphical summaries of the relationship between *x*_*i*_ and *y* for fitted regression models: The plot calculates the response for each predefined value of *x*_*i*_ with the remaining *p*-1 variables fixed, i.e., $\hat y = e^{\beta _0 + \beta _1\bar x_1 + \ldots + \beta _ix_i + \ldots + \beta _p\bar x_p}$, where $\bar x_p$ denotes the mean of *x*_*p*_.

To enable automatic detection and learning of nonlinear relationships and interactions, ML incorporates complex model structures that require advanced interpretation tools to ensure transparency [23, 37]. The Shapley value is employed here, because (a) it is a tool providing local “granular” metrics which can be “rolled up” to less granular metrics for implementing various interpretation tasks (feature importance, effect trend, and interaction plot, etc.), and (b) it is based on solid theoretical foundations (coalitional game theory) providing a unique solution satisfying properties (local accuracy, consistency, and missingness) desired for explanatory ML analysis [38].

The Shapley value for the *i*th variable *φ*_*i*,*j*_ measures the contribution to the prediction of the *j*th sample, calculated as the average of the marginal responses over all possible coalitions of the remaining *p*-1 variables. The prediction of the *j*th sample satisfies (additivity property): ${{{{{{{\mathrm{log}}}}}}}}(y_j) = \varphi _0 + \varphi _{1,j} + \ldots + \varphi _{i,j} + \ldots + \varphi _{p,j}$, where *φ*_0_ is a constant representing the prediction average across all samples. For fair comparison, we similarly defined the “Shapley effects plot” for variable *i* through estimated responses for all samples, each with the remaining *p*-1 Shapley values (excluding *φ*_*i*,*j*_) fixed, i.e., $\hat y_j = e^{\varphi _0 + \bar \varphi _{1,j} + \ldots + \varphi _{i,j} + \ldots + \bar \varphi _{p,j}}$, where $\bar \varphi _{p,j} = \frac{1}{n}\mathop {\sum}\nolimits_{j = 1}^n {\varphi _{p,j}}$. The relative percent changes of $\hat y_j$ for the first and third quartile of *x*_*i*_ with respect to the median were calculated and their average was used to quantify the associations. It should be noted that this metric may become invalid for highly nonlinear patterns as, for instance, positive and negative associations can be canceled in a non-monotonic trend.

### Change of support validation

Two hundred nine NJ municipalities did not make COVID-19 death data available and their numbers were predicted with the eight base models; all estimates were aggregated by county to compare with the county-level deaths reported by the NJDOH. Though the focus of this study is on effect analysis (i.e., magnitude and significance of regression coefficients) for socioexposomic health studies, prediction results (i.e., responses of models) on other scales (change of support), where observations are available, can provide additional insight into the reliability of different analytical models.

### Simulation analysis

Simulation analysis was performed to improve comparisons of association estimates obtained from different approaches. Geostatistical and machine learning models were constructed using simulated data generated for three representative scenarios (see Text S4).

#### Socioexposome generation

To maintain realistic correlation information (linear/nonlinear), we set the “simulated inputs” *X* as the actual socioexposomic data; for simplicity, we did not consider the uncertainty of socioexposomic variables.

#### Health outcome generation

To simulate “responses” with overdispersion, group randomness and spatial autocorrelation, we generated health outcomes *Y* as a function of the socioexposome:

1 $$Y 	 \sim {{{{{\rm{NBinomial}}}}}}(\lambda ,\theta ) \\ \log \left( \lambda \right)	 = \mathop {\sum}\nolimits_{i = 1}^{10} {\beta _iX_i + v + u + {{{{{{{\mathrm{log}}}}}}}}({{{{{\rm{population}}}}}})}$$

In Eq. (1), municipality death number *Y* is drawn from Negative-Binomial distribution with mean *λ* and a size parameter *θ* that controls the overdispersion strength. log(*λ*) equals the summation of three components plus an offset term (i.e., logarithm of municipality population). The fixed effect component $\mathop {\sum}\nolimits_{i = 1}^{10} {\beta _iX_i}$ was calculated as a linear combination of ten selected socioexposomic variables (true predictors). The ordinary random effect component *v* follows a univariate normal distribution $N(0,\sigma _v^2)$, where $\sigma _v^2$ denotes the variance of the random effect/intercept. The spatial random effect component *u* follows a multivariate normal distribution (Gaussian process) *MVN*(**0, Σ**), where ${{{{{{{\mathbf{\Sigma }}}}}}}} = \sigma _u^2({{{{{{{\mathbf{I}}}}}}}} - \rho {{{{{{{\mathbf{W}}}}}}}})^{ - 1}$ represents the spatial correlation matrix, $\sigma _u^2$ denotes the conditional variance of the spatial effect, *ρ* denotes the spatial coefficient, and **W** represents the adjacency matrix.

#### Simulation scenarios and parameter settings

Without loss of generality, regression coefficients of the ten “true predictors” were set as: *β*_0_ = −5, *β*_1_ = 0.5, *β*_2_ = 0.35, *β*_3_ = 0.2, *β*_4_ = 0.2, *β*_5_ = 0.25, *β*_6_ = 0.05, *β*_7_ = 0.15, *β*_8_ = −0.05, *β*_9_ = 0.15, *β*_10_ = −0.5. **W** was calculated based on the neighbor relationships of the 565 municipalities. We set *θ* = 20 to simulate significant overdispersions and *ρ* = −0.2 to ensure **Σ** positive definite.

We considered three scenarios based on two metrics: *r*_1_ measures the proportion of variance explained by predictors among the total fixed/random effect components and *r*_2_ the proportion of variance explained by the spatial random effect in the total random components. Scenario 1 (*r*_1_ = 0.6, *r*_2_ = 0.6) is defined as a reference scenario; Scenario 2 (*r*_1_ = 0.6, *r*_2_ = 0.3) corresponds to more significant ordinary random effect; Scenario 3 (*r*_1_ = 0.3, *r*_2_ = 0.6) corresponds to more significant total random effect. $\sigma _v^2$ and $\sigma _u^2$ determine *r*_1_ and *r*_2_, and the relevant parameters and simulated distributions are presented in Fig. S8. Furthermore, we investigated the impact of missing data on association estimation by constructing spatial models using a subset of simulated data where the sample size equals that of the realistic case.

## Results

COVID-19 morbidity and mortality were found to be correlated with a range of socioexposomic factors, among which strong intercorrelations were also observed (Fig. S3). The spatial distribution of the LTCF deaths across New Jersey is depicted in Fig. S2. By removing LTCF deaths, the Spearman correlation coefficient (*ρ*) between COVID-19 death rates and % uninsured increased from 0.27 to 0.38; for % below high school education, the correlation increased from 0.24 to 0.36. However, exclusion of LTCF deaths does not significantly affect correlations (|Δ*ρ*| < 0.08) for other correlated factors (*ρ* > 0.2). Similar results hold for the case rates.

Table S4 presents the associations of municipality COVID-19 death rates with different factors estimated from eight models. The Poisson regression model provided the narrowest 95% confidence intervals (CIs) for all variables. The Poisson mixed effect model improved the outcomes by considering randomness from five geographical areas, with a lower DIC metric (Table S2). However, inclusion of the random effect component (i.e., the NB mixed effect model) does not affect substantially the outcomes of the NB regression model. The Poisson/NB BYM spatial models provided the widest CIs due to the simultaneous consideration of random effect and spatial autocorrelation. The RF and XGBOOST models are deterministic and do not provide CIs.

**Fig. 2:**
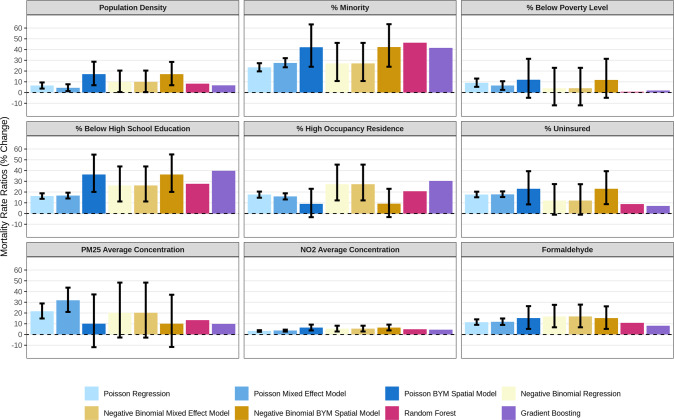
Associations of municipality COVID-19 mortality rates with 9 selected socioexposomic factors, calculated from 8 geostatistical and machine learning models. The black vertical lines depict the 95% confidence intervals (CIs) of the estimates.

Figure 2 shows the associations of COVID-19 deaths rates with 9 selected factors estimated from the eight models. All eight models revealed significant positive association of COVID-19 death rates with population density: the Poisson/NB BYM spatial models indicated that 20% quantile increase in population density is associated with 17% (95% CI: 7%, 29%) increase in mortality rate and the ML models indicated 7–8% (RF and XGBOOST estimates) increase in mortality rate. For % below high school education, the BYM spatial models indicated that a standard deviation increase (6.3%) is associated with 36% (20%, 55%) increase in mortality rate and the ML models indicated 28–40% increase in mortality rate. For NO_2_ average concentration, the BYM spatial models indicated that 1 ppb increase is associated with 6% (4%, 9%) increase in mortality rate and the ML models indicated 4–5% increase in mortality rate. Association analyses for other socioexposomic factors are presented in Text S3. To facilitate interpretation, some variables were normalized and the units are specified in Table S4.

**Fig. 3:**
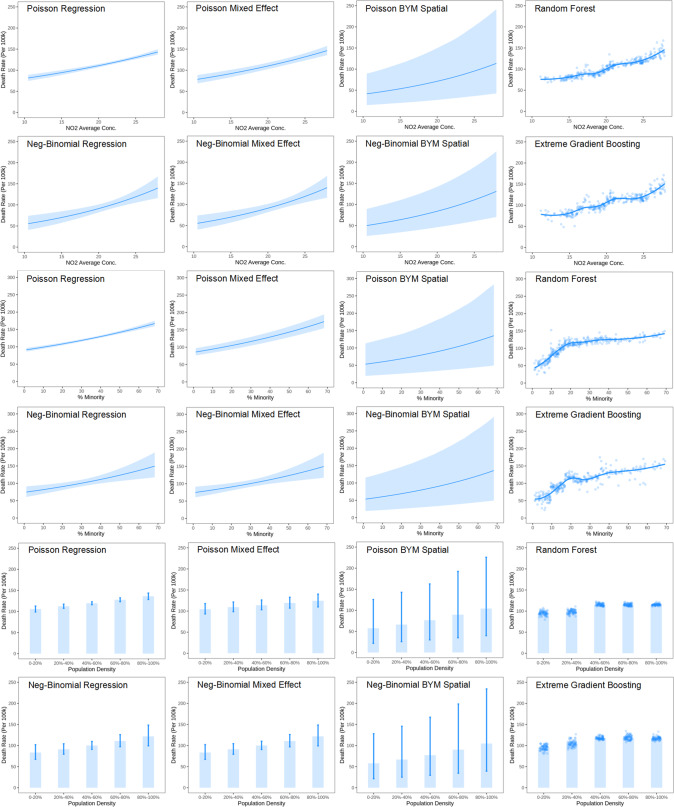
Effects plots of 3 representative socioexposomic factors from 8 geostatistical and machine learning models. 1st-2nd rows: NO_2_ average concentration, 3rd-4th rows: % minority, 5th-6th rows: population density.

The effects plots for the 9 selected factors estimated from the eight models are displayed in Fig. S7. The different models captured consistent trends, though differences exist in the description of complex patterns. The six geostatistical models only described the exponential relations, while the two ML models can capture more complex relations. The nonlinear effects captured by RF and XGBOOST are similar and stable, pointing to the presence of underlying nonlinear relations in the data (Fig. 3). The ML models detected an exponential increasing trend of mortality rate for NO_2_ average concentration, which is consistent with the assumption of the geostatistical models, thus leading to similar association estimates. In addition, the ML models “learned” a steeper increasing trend for % minority below 20%; this corresponds to a situation when the exponential assumption is violated. The ML models also “learned” that COVID-19 mortality rates increase with population density at the lower range but become “saturated” at higher densities [34], a fact that cannot be captured by geostatistical models with “naive” structures.

**Fig. 4:**
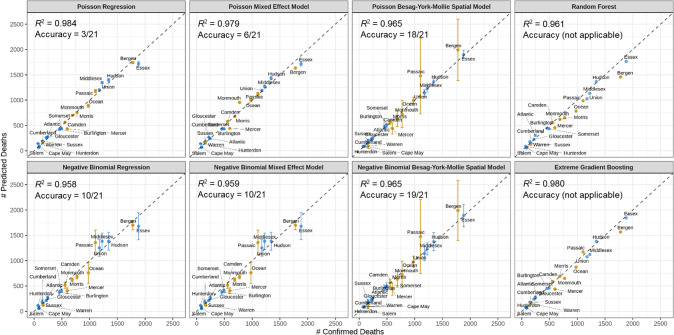
Scatter plots of the observed vs predicted county COVID-19 death numbers calculated from 8 geostatistical and machine learning models. The blue points represent counties where all municipalities have deaths reported and the yellow points represent counties with unavailable deaths in at least one municipality. The vertical lines depict the 95% CIs of the predictions and accuracy measures the proportion of predictions where the CIs contain the observed values.

**Fig. 5:**
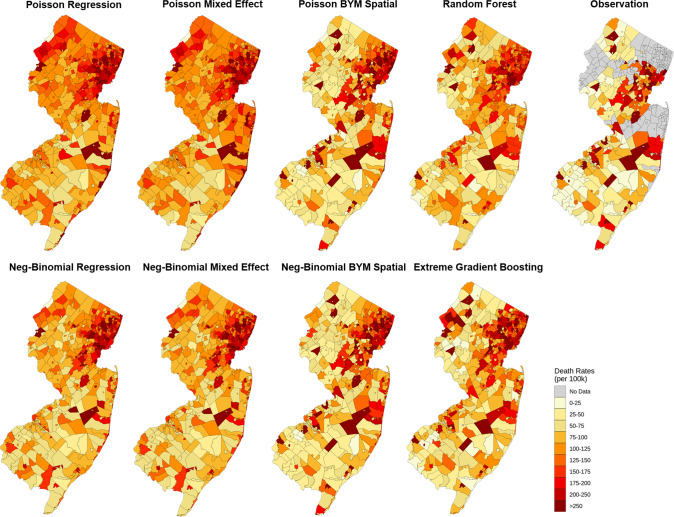
Spatial distributions of the predicted and observed municipality COVID-19 mortality rates (at the end of the first phase of the pandemic in New Jersey, September 24, 2020) calculated from 8 geostatistical and machine learning models. The first three columns correspond to 6 geostatistical models, the fourth column corresponds to 2 machine learning models, and the last column corresponds to observations.

Figure 4 presents the change of support validation results for the eight models in estimating county COVID-19 death numbers during the first wave in New Jersey. The two ML models achieve prediction accuracy comparable to all six geostatistical models considered. Though the Poisson regression model achieved the highest *R*^2^ (=0.984), the estimated 95% prediction intervals (PIs) are unreliable (Accuracy = 3/21). In comparison, the BYM spatial models can capture uncertainties and variations well, with the lowest DIC metric (Table S2) across the six geostatistical models. In the NB BYM spatial model, 19 out of 21 PIs contain the exact values (Accuracy = 19/21). In addition, the BYM spatial models capture local spatial variations better than Poisson and NB regression (Fig. 5). Though the ML models do not provide CIs, their ability to capture spatial variations was comparable to that of the BYM spatial models.

**Fig. 6:**
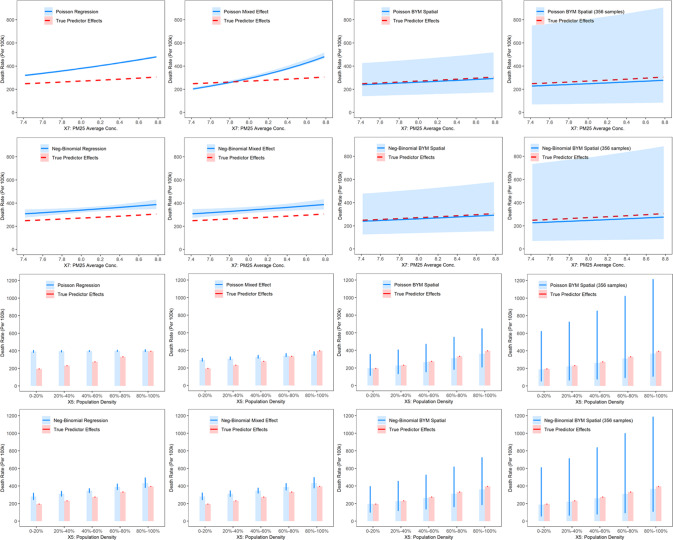
Predictor effects plots of 2 representative socioexposomic factors from different geostatistical models in a reference simulation scenario. The first three columns correspond to models fitted to 565 data samples, and the last column corresponds to models fitted to 356 data samples.

Figure 6 shows simulation outcomes comparing true and estimated predictor effects profiles of two selected variables from eight geostatistical models in a reference scenario. The BYM spatial models (column 3 of Fig. 6) produced estimates closer to the truth; the remaining four statistical models (columns 1–2 of Fig. 6) exhibited significant biases, with the true predictor effects profiles being outside the 95% CIs. Missing data do not drastically affect the association estimates but increase uncertainties for spatial models (column 4 of Fig. 6): such uncertainties will decrease for a smaller proportion of variance explained by the spatial effect in the data (columns 1-2 of Fig. S10). In the simulation study, we did not observe performance of ML comparable to advanced geostatistical models, while ML successfully detected the exponential trend and generated slopes (association strengths) consistent with the truth (Fig. S11). Simulation outcomes, including regression coefficients (Tables S7-S9) and predictor effects profiles (Fig. S9), for the remaining variables for each of the three scenarios are available in the Supplement (Text S4).

## Discussion

Efficient modeling tools are needed for improved, interpretable, socioexposome-wide association studies. To our knowledge, this is the first work that conducted a comprehensive comparison of different geostatistical and ML approaches to model associations of multiple socioeconomic, demographic and environmental factors with COVID-19 outcomes.

Various regression models have been employed in previous association studies of COVID-19 outcomes with social and environmental factors, but caution should be exercised in interpreting such analyses [26]. The present study shows that estimated associations with health effects can be substantially influenced by model structures and assumptions, and a reliable estimation should be based on frameworks that ensure adequate considerations of data behaviors. Among the factors considered, the estimated CIs for the Poisson regression model were 9–41% (mortality rates) narrower than those for the BYM spatial models (corresponding to lower *p*-values), due to insufficient description of overdispersion. The NB regression and mixed effect models provided wider CIs than the Poisson regression model; however, their point estimates deviated by −14% to 14% from those of the BYM spatial models. The Poisson/NB BYM spatial models provided the “best” association estimates with the lowest DIC metric; both approaches modeled significant variations from overdispersion, group randomness, and spatial autocorrelation (*p* < 0.05). Regarding prediction (change of support validation), the BYM spatial models can identify reasonably higher uncertainties for areas with clustered missing data, while the other models provided narrow PIs that were overly optimistic (Fig. 4).

Simulation analysis was performed to evaluate and interpret association estimates, enabling a direct comparison with “true” benchmarks. Previous simulation studies used ordinary linear regression to generate data for detecting “true” variables and interactions [20–22]. The present study implemented a simulation method appropriate for complex data variations that strengthened the evidence regarding insufficient description of data variability causing significant biases, while missing data increasing uncertainties of association estimates. Simulations also improved interpretation of modeling results: statistical models such as Poisson regression are simple but significantly biased; advanced geospatial models are more accurate but somewhat conservative in the case of missing data; machine learning, while not superior in modeling linear data, can capture underlying complex patterns. It is therefore advisable to compare multiple models that can help compensate for the shortfalls of some methods over others.

The feasibility and effectiveness of both Frequentist and Bayesian frameworks were investigated in model development. Most previous models were built using Frequentist frameworks [3–5, 7] while Bayesian frameworks can consider flexible uncertainties in model parameters and incorporate prior expert knowledge with hierarchical structures [36]. Herein, Frequentist inference for the six geostatistical models (i.e., generalized, mixed effect and BYM spatial Poisson/NB regression) was implemented with three R packages (stats, lme4 and spaMM) and Bayesian inference for the six models was implemented with R-INLA (Integrated Nested Laplace Approximation) [39]. By setting non-informative priors, Bayesian inference obtained results almost identical to those from Frequentist inference, with <1% mortality rate errors across all association estimates (Table S5); however, Bayesian inference was more computationally efficient than Frequentist inference, especially for complex models. For example, it takes 9.02 ± 0.53 s (Intel Xeon 6130) to build the Bayesian NB BYM spatial model, while 60.63 ± 2.50 s are required for Frequentist inference (Table S6). Frequentist inference must solve optimization problems and the computational load increases rapidly for complex structures with additional parameters to be estimated; Bayesian inference gains enhanced computational efficiency through the Laplace approximation implemented in R-INLA. Because of their higher flexibility and efficiency, Bayesian frameworks provide advantages over Frequentist frameworks for ecological and environmental health modeling.

Comprehensive comparisons of geostatistical and ML models for exposomic health association studies have been rare. Here, we extended the predictor effects plot, and derived a generic Shapley effects plot (different from the Shapley scatter plot) to perform fair comparisons in health effect analysis. An association metric was also constructed to approximate effects captured by the ML models and showed that ML (even for small datasets) played a complementary role to advanced geostatistical models: those models can capture similar associations when an underlying exponential relation holds, but ML can further “learn” non-exponential patterns in the data, an attribute that is important for knowledge discovery. For instance, RF and XGBOOST detected nonlinear saturation effects of increasing population density that can be explained by the combined effects of “density-dependent” and “frequency-dependent” mechanisms [34, 40].

Applications of ML on large datasets (e.g., over 100,000 samples) have been evaluated in environmental health modeling [41, 42]. For small/moderate sample size (e.g., tens or hundreds of samples), earlier studies found no evidence of superior performance of ML over traditional statistical models [21, 43]. ML was considered “data hungry” [44], and in fact, instability and overfitting are prone to occur when ML models are trained with small sample sets. These problems are exacerbated for data with large errors: ML tends to treat random noise as nonlinear patterns, so an overinterpretation among small samples can lead to poor generalization and unstable results. In contrast, geostatistical models employ “naive” structures that inherently facilitate robustness with respect to data noise. To avoid overfitting and to stabilize model performance, different ML hyperparameters must be tuned carefully using multiple evaluation metrics to control model complexity and learning capability [23]. Furthermore, explainability (interpretability) testing and model refinement are essential for obtaining reliable ML models applicable to effect characterization and prediction [45].

Associations obtained from the complementary modeling frameworks strengthen the evidence on the role of multiple environmental and social determinants on COVID-19 severity. Our estimate for NO_2_ effect size is similar across the eight models considered, and it is consistent with the Los Angeles County neighborhoods study [46], that observed a 5.6% (3.7%, 7.5%) increase in mortality rate for every 1 ppb increase in NO_2_. Ours and Lipsitt’s local studies, accounting for greater spatial variability, detected larger effect size than that reported for a US nationwide county-level study [3], that observed a 3.3% (1.8%, 4.8%) increase in mortality rate for every 1 ppb increase in NO_2_. We did not observe stable significant positive association between COVID-19 death rates and annual PM_2.5_ average concentration (Table S4), while ML models identified a positive nonlinear association for annual PM_2.5_ averages above 8 μg/m^3^ (Fig. S7). We found that a small increase in concentrations of certain air toxics can lead to large increases in mortality rate, which is in alignment with the US nationwide county-level study of Petroni et al. [5]. We observed significant positive associations between mortality rate and proximity to industrial facilities and waste sites (traffic, NPL sites, TWWD sites, and TSDF facilities). Transportation-related noise was highly correlated with mortality rate (*ρ* = 0.43); however, the association adjusted with confounders was statistically insignificant [18]. We observed a significant negative association of mortality rate with the 15–44 age group and significant positive association with age group >64, which is consistent with the J-shaped nature of the age pattern for COVID-19 mortality [47]. We also found that multiple demographic and socioeconomic factors were significantly associated with mortality rates: these patterns can be considered as driving forces associated with racial/ethnic and socioeconomic disparities [7] of the pandemic across New Jersey.

Our study has limitations: First, associations were obtained from an ecological design that may not reflect individual associations [26]. Such biases are relevant to data gaps that cannot be addressed by solely improving analytical tools; however, to mitigate the issue, the study accounted for “sub-county” spatial variations (for areas that are more homogeneous with respect to socioexposomic factors than counties) and is the first work that conducted comprehensive socioexposomic analyses for COVID-19 at municipality scale across a State. Second, we did not study synergistic effects of stressor/exposure “mixtures”, though ML actually detected interactions that can be directly interpreted with the Shapley value. Like the exposure-response cross-section function in Bayesian kernel machine regression (BKMR) [48], Shapley values can provide an interaction analysis tool for arbitrary ML models. RF and XGBOOST (tree ensemble algorithm) were considered here, due to their high computational efficiency and learning capability [23]. Third, the ML models in the present study employed a deterministic framework and therefore did not provide CIs/PIs that can be important in health effect analyses.

Our regional socioexposomic study revealed significant associations between COVID-19 health outcomes and multiple, spatially heterogeneous, stressors: this evidence can help improve the understanding of social and environmental justice issues relating to the impact of the pandemic. Integrated methodological frameworks were developed, to examine stability and complex behaviors of factor associations. Our results suggest that the Bayesian geospatial models have advantages over the Frequentist statistical models in precision health/exposomic modeling. Furthermore, explainable (interpretable) ML/AI can be an effective supplement to traditional geostatistical modeling for uncovering underlying complex patterns, even for small/moderate data sets. The tools and analyses presented here can be extended to other ecological and individual-level health studies at multiple scales.

### Supplementary information

Supplementary Material

## Data Availability

All data analyzed in this article are publicly available at https://github.com/ccl-group/Data-for-JESEE-22-3920.
