# Supplementary material for: Socioexposomics of COVID-19 across New Jersey: a comparison of geostatistical and machine learning approaches
Source: J Expo Sci Environ Epidemiol. 2023 Feb 1;34(2):197–207. doi: 10.1038/s41370-023-00518-0 (PMC9889956; doi:10.1038/s41370-023-00518-0)
Supplement: Supplementary file 1 — Supplementary Material [file 41370_2023_518_MOESM1_ESM.doc]

**SUPPLEMENTARY MATERIAL**

Socioexposomics of COVID-19 across New Jersey: a comparison of geostatistical and machine learning approaches

Xiang Ren^1,2^, Zhongyuan Mi^1,3^ and Panos G. Georgopoulos1,2,3,4[[1]](#footnote-2)*

^1^ Environmental and Occupational Health Sciences Institute (EOHSI), Rutgers University, Piscataway, NJ 08854, USA

^2^ Department of Chemical and Biochemical Engineering, Rutgers University, Piscataway, NJ 08854, USA

^3^ Department of Environmental Sciences, Rutgers University, New Brunswick, NJ 08901, USA

^4^ Department of Environmental and Occupational Health and Justice, Rutgers School of Public Health, Piscataway, NJ 08854, USA

**Text S1. Additional information on data sources**

*COVID-19 cases/deaths.* Cumulative COVID-19 cases/deaths as of September 24, 2020 were obtained from local health departments across New Jersey. We were able to collect case data for 563 and death data for 356 from the 565 municipalities in New Jersey. Deaths in long-term-care facilities (LTCF) were provided by NJDOH, and these numbers were subtracted from total deaths in each municipality in order to consider mortality rates calculated with and without LTCF deaths in correlation analyses. Since in the early phases of the pandemic testing was inadequate, the analytical models used death (instead of case) data to ensure higher credibility.

*Socioeconomic status.* Socioeconomic factors considered here included education, language isolation, household crowding, poverty, disability, unemployment, uninsured community, social vulnerability index (SVI), etc. Individual socioeconomic variables were available at municipality resolution directly from ACS; SVI estimates were calculated using the 15 individual socioeconomic variables across all NJ municipalities, applying the same methodology used by CDC ([https://www.atsdr.cdc.gov/placeandhealth/svi/ index.html](https://www.atsdr.cdc.gov/placeandhealth/svi/index.html)).

Effects of both individual variables and combined indices on COVID-19 outcomes were considered in the correlation analysis. For association quantification, base models were constructed via a variable selection algorithm that started with essential individual variables, while associations of the remaining highly correlated individual variables and/or indices were estimated separately.

*Air pollutants.* Metrics for three criteria air pollutants were considered in this study: Annual average PM_2.5_ and summer seasonal average of daily maximum 8-hour ozone concentrations (2016) were retrieved from the EJSCREEN at the block group level. Annual averages of daily maximum 1-h NO_2_ concentrations (2016) were made available by Di et al. [1] at 1×1 km^2^ resolution. These estimates were then assigned to municipalities via population weighted averaging.

Twenty air toxics were selected based on their respiratory hazard quotient (HQ) values from the EPA NATA for 2014. Two indices characterizing combined effects of air toxics on health risks were also considered, i.e., respiratory hazard index and lifetime inhalation cancer risk. Five specific air toxics (formaldehyde, acetaldehyde, acrolein, naphthalene, and diesel PM), accounting for over 50% of the total US respiratory hazard index [2] were studied in the analytical models.

**Text S2. Development of statistical models: Frequentist inference vs Bayesian inference**

In the Frequentist framework, it is assumed that there exist underlying “true” values of model parameters, so in statistical inference, point estimates of the exact values are optimized by maximizing the likelihood function while confidence intervals (ranges containing the exact values for a given frequency) are approximated using Gaussian assumptions. In contrast, the Bayesian framework assumes model parameters to be random variables that are described by hierarchical distributions with prior knowledge (non-Gaussian). The posterior distributions are simulated to provide arbitrary summary statistics such as posterior mean and credible intervals (ranges within which the unobserved parameter values fall with a given probability).

**Text S3. Association analysis for other socioexposomic factors**

As expected, all eight models showed significant negative association of COVID-19 death rates with % population in the age range 15-44 and significant positive association with % population of age > 64, while associations for the other two age groups (age < 15 and age 45-64) were statistically insignificant (Fig. S4). All eight models revealed significant positive association of COVID-19 death rates with % population (Asian) and % population (Hispanic) and significant negative association with % population (White) (Fig. S5). All eight models revealed significant positive association of COVID-19 death rates with four environmental proximity factors, NPL site proximity, TSDF facility proximity, proximity to TWWD and traffic proximity (Fig. S6).

**Text S4. Simulation Study**

*Generating simulated data.* In contrast to previous exposome simulation studies selecting true predictors [3] or detecting true interactions [4, 5] across different (linear) statistical methods, the present simulation study aims to evaluate and compare the performance of different geostatistical and (nonlinear) machine learning models for characterization and quantification of exposomic associations. For simplicity, our simulation analyses were performed with ten variables selected in the base model, which include *X*_1_: % population (age > 64), *X*_2_: % minority, *X*_3_: % below high school education, *X*_4_: median gross rent, *X*_5_: population density, *X*_6_: % occupation (high risk), *X*_7_: PM_2.5_ average concentration, *X*_8_: ozone seasonal DM8HA, *X*_9_: % high occupancy residence, and *X*_10_: % unemployed.

The actual exposome data were directly used as the simulated inputs, given that assessing effect of input uncertainties on prediction performance was not the focus in the present study. However, to further consider such uncertainties, we recommend using bootstrap resampling from the actual exposome data to maintain realistic correlation structure. It should be noted that our socioexposomic factors contain percentage variables that range from 0 to 100, so sampling methods used in previous exposome simulation studies (i.e., drawing from multivariate normal distribution [3, 4] or multivariate log-normal distribution [5]) would be inappropriate.

A low-level multi-step sampling approach was introduced to generate health outcome *Y* (i.e., municipality COVID-19 death number) exhibiting overdispersion, group randomness and spatial variation, as formulated in Eq. 1:

$\begin{matrix} \log(\lambda)=\sum_{i=1}^{10} \beta_{i}\cdot X_{i}+v+u+\log(\text{population}) \\ Y\sim\text{NBinomial}(\lambda， \theta) \end{matrix}$ (1)

For Step 1, the fixed effect component $\sum_{i=1}^{10} \beta_{i}\cdot X_{i}$ was calculated as a linear combination of ten socioexposomic variables, where $\beta_{i}$ denotes the regression coefficient of the *i*^th^ variable *X*_i_. For Step 2, the ordinary random effect component *v* was simulated by sampling from a univariate normal distribution $N(0, \sigma_{v}^{2})$, where $\sigma_{v}^{2}$ denotes the variance of the random effect/intercept. For Step 3, the spatial random effect component was simulated by sampling from a multivariate normal distribution $MVN(0, \mathbf{\Sigma})$, where $\mathbf{\Sigma}=\sigma_{u}^{2}(\mathbf{I}-\rho\mathbf{W})^{-1}$ is the spatial correlation matrix, $\sigma_{u}^{2}$ is the conditional variance of the spatial effect, *ρ* is the spatial coefficient, and **W** is the adjacency matrix corresponding to the overall 565 New Jersey municipalities. For Step 4, the conditional mean *λ* was calculated by summing up all components simulated from Steps 1-3 plus an offset term (logarithm of municipality population). For Step 5, the health outcome *Y* was generated via a Negative-Binomial distribution with mean *λ*, where the size parameter $\theta$ was used to control overdispersion strength in the count data *Y*.

*Simulation scenarios and parameter settings.* Without loss of generality, regression coefficients were set as $\beta_{0}=-5$, $\beta_{1}=0.5$, $\beta_{2}=0.35$, $\beta_{3}=0.2$, $\beta_{4}=0.2$, $\beta_{5}=0.25$, $\beta_{6}=0.05$, $\beta_{7}=0.15$, $\beta_{8}=-0.05$, $\beta_{9}=0.15$, $\beta_{10}=-0.5$. In the spatial effect component, the spatial coefficient was set as $\rho=-0.2$, the adjacency matrix was generated based on the realistic neighbor relationships among the 565 New Jersey municipalities, as described in the main manuscript. For the overdispersion effect, the size parameter was set as $\theta=20$.

We considered three scenarios based on two variance-related ratio metrics as defined below: *r*_1_ measures the proportion of variance explained by predictors among the total variance of the fixed and random components, *r*_2_ measures the proportion of variance explained by the spatial random effect among the total variance of the random components.

$\begin{matrix} r_{1}=\frac{\mathrm{var}(\sum_{i=1}^{10} \beta_{i}X_{i})}{\mathrm{var}(\sum_{i=1}^{10} \beta_{i}X_{i})+\mathrm{var}(v)+\mathrm{var}(u)} \\ r_{2}=\frac{\mathrm{var}(u)}{\mathrm{var}(v)+\mathrm{var}(u)} \end{matrix}$ (2)

In Eq. 2, $\mathrm{var}(v)=\sigma_{v}^{2}$, var(*u*) is determined by $\sigma_{u}^{2}$, *ρ* and **W**. In the present study, var(*u*) is found to be close to $\sigma_{u}^{2}$. Scenario 1 $(r_{1}=0.6, r_{2}=0.6)$ corresponds to the reference scenario. Scenario 2 $(r_{1}=0.6, r_{2}=0.3)$ aimed to assess the impact of spatial effect on association quantification, where the ordinary (municipality-specific) random effect dominates in the total random effects. Scenario 3 $(r_{1}=0.3, r_{2}=0.6)$ investigated the impact of total random effects on association quantification, where the total random effects dominate in the total effects. 565 samples corresponding to the overall 565 New Jersey municipalities were generated for each of the three scenarios. To satisfy the constraints on *r*_1_ and *r*_2_, we defined $\sigma_{v}^{2}=0.18, \sigma_{u}^{2}=0.25$ for Scenario 1, $\sigma_{v}^{2}=0.30, \sigma_{u}^{2}=0.12$ for Scenario 2, and $\sigma_{v}^{2}=0.65, \sigma_{u}^{2}=0.90$ for Scenario 3. Figure S8 shows the density plot of 565 samples drawing from the ordinary (municipality-specific) random effect distribution and the spatial (conditional autoregressive structural) random effect distribution for each of the three scenarios. The remaining parameters as defined above were kept the same over the three scenarios. Furthermore, we investigated the impact of missing data on socioexposomic association estimates by fitting the spatial models to a specific subset (356) of the simulated data; that subset corresponds to the 356 municipalities with available death data in the real case.

*Simulation analysis.* Tables S7-S9 present the true regression coefficients of 10 socioexposomic variables and the corresponding estimates from eight statistical and geospatial models for three scenarios. All these scenarios indicate that Poisson/Negative-Binomial BYM spatial models can produce the best estimates for regression coefficients (closest to the true values). This is explainable because (a) Negative-Binomial BYM spatial model uses a structure similar to the low-level multi-step sampling approach that generates the data (Eq. 1), and (b) Poisson BYM spatial model involves a flexible structure that can also describe the overdispersion behavior well, with the ordinary and spatial random effect components. Without fully considering potential random effects, Poisson regression performed the worst (with the largest deviations from the true values and none of the 95% confidence intervals of the 10 variables contained the true values); furthermore, the performance deteriorated with decreasing proportion of variance explained by predictors (Scenario 1 vs Scenario 3).

Figure S9 depicts the effects plots of 10 variables from 8 statistical and geospatial models for Scenario 1 (reference scenario). For Poisson/Negative-Binomial regression and Poisson/Negative-Binomial random effect models, none of the 95% prediction intervals contain the true predictor effects profiles. Poisson/Negative-Binomial BYM spatial models can produce predictor effects profiles close to the true curves: The point estimates were almost unchanged considering missingness of data (4^th^ column of Figure S9), while spatial models fitted to 356 data produced wider CIs compared to those fitted to 565 data. Figure S10 compares the predictor effects plots of X_7_ from Poisson BYM spatial model for three scenarios, fitted to 565 samples and 356 samples respectively. It shows that CIs incorporating data missingness become wider when the proportion of variance explained by the spatial effect increases (Scenario 1 vs Scenario 2). Similar results were observed for other variables (Table S7 vs Table S8).

Figure S11 shows the Shapley effects plots of 10 variables from Random Forest. The Machine Learning model performed worse than geostatistical models in the simulation study, with larger deviations from the true predictor effects profiles. Though such deviations can be partly due to the fact that predictor effects plot (used for statistical modeling) and Shapley effects plot (used for machine learning) are not strictly equivalent. It should be pointed out that machine learning is able to automatically detect the underlying trends (herein, exponential for the simulated data) and generate slopes (association strengths) basically consistent with the true slopes.

Simulation studies can provide valuable information for interpretation, but they are insufficient to identify a “best” model that can be directly applied to an actual case, due to the complexity of the real-world data. It is expected that advantages of ML will become more prominent for modeling data with large proportion of variance explained by highly nonlinear patterns, which requires further investigation of advanced nonlinear simulation techniques. Due to the unavailability of death data in the 209 municipalities, Poisson/Negative-Binomial BYM spatial models were expected to produce conservative association results (wider CIs). To improve association estimation, it is advisable to compare multiple models that can help compensate for the shortfalls of some methods over others.


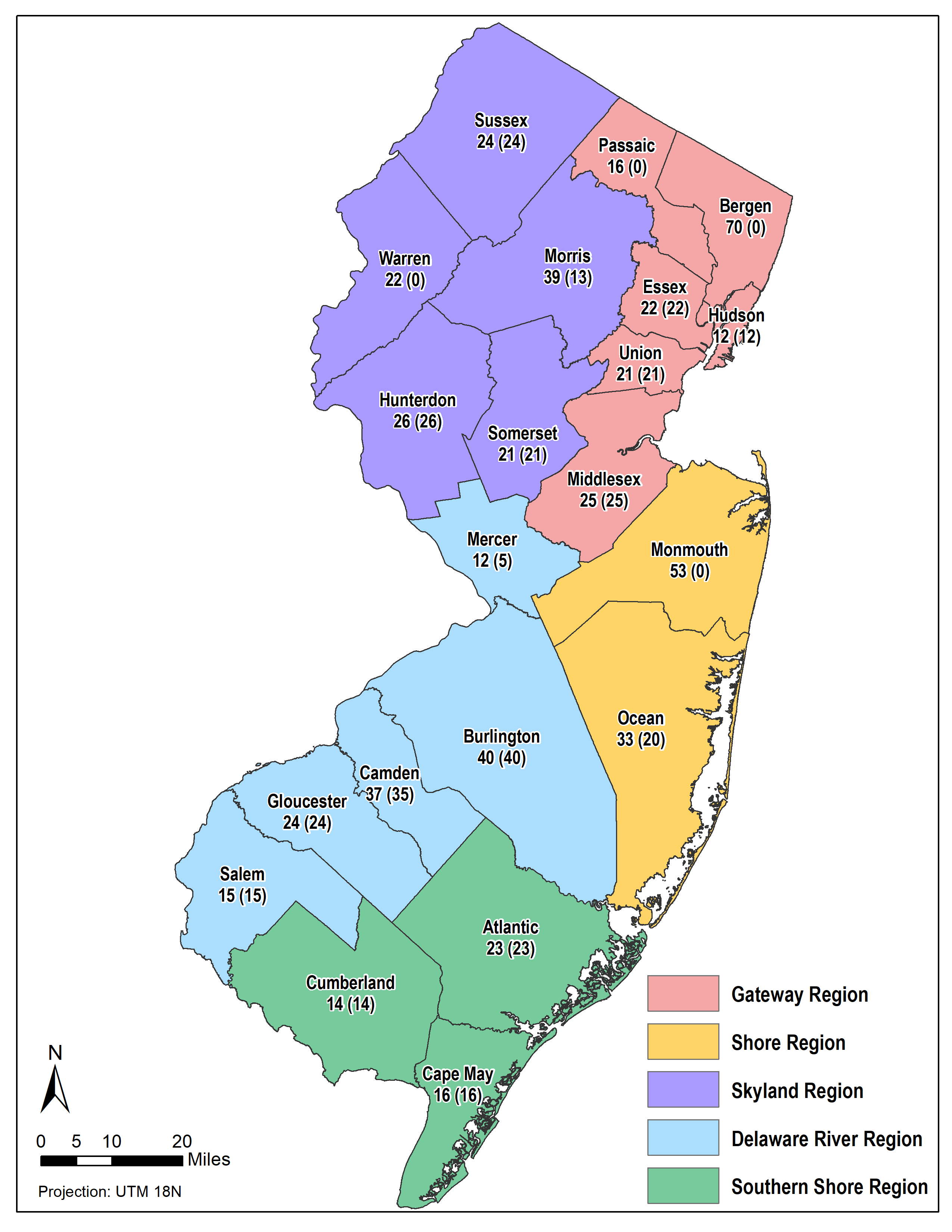


**Fig. S1** The 21 Counties and the five geographical regions identified based on similarities of exposure-relevant factors across the State of New Jersey. The total number of municipalities in each county is shown outside the parenthesis; the numbers inside the parenthesis are the municipalities with available death data.


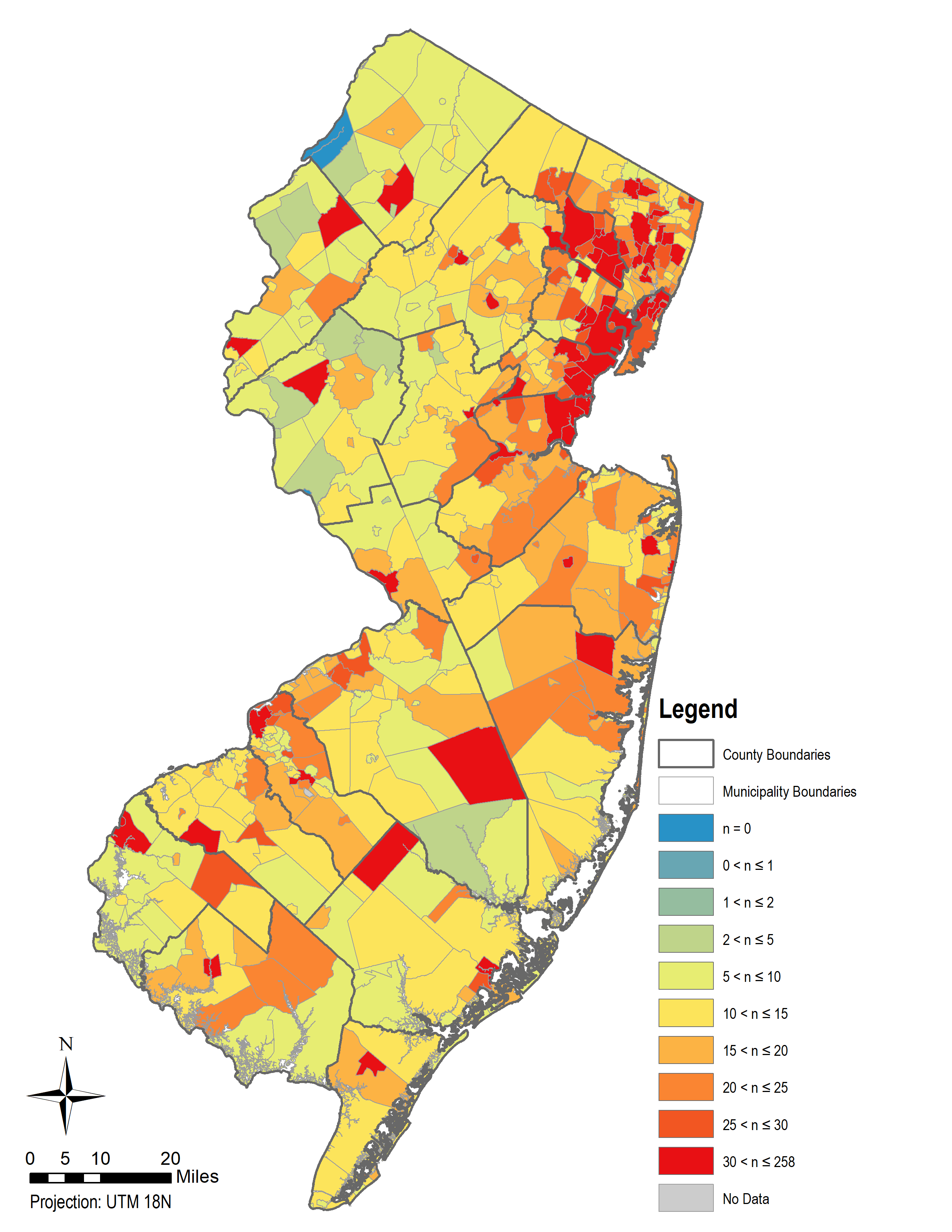

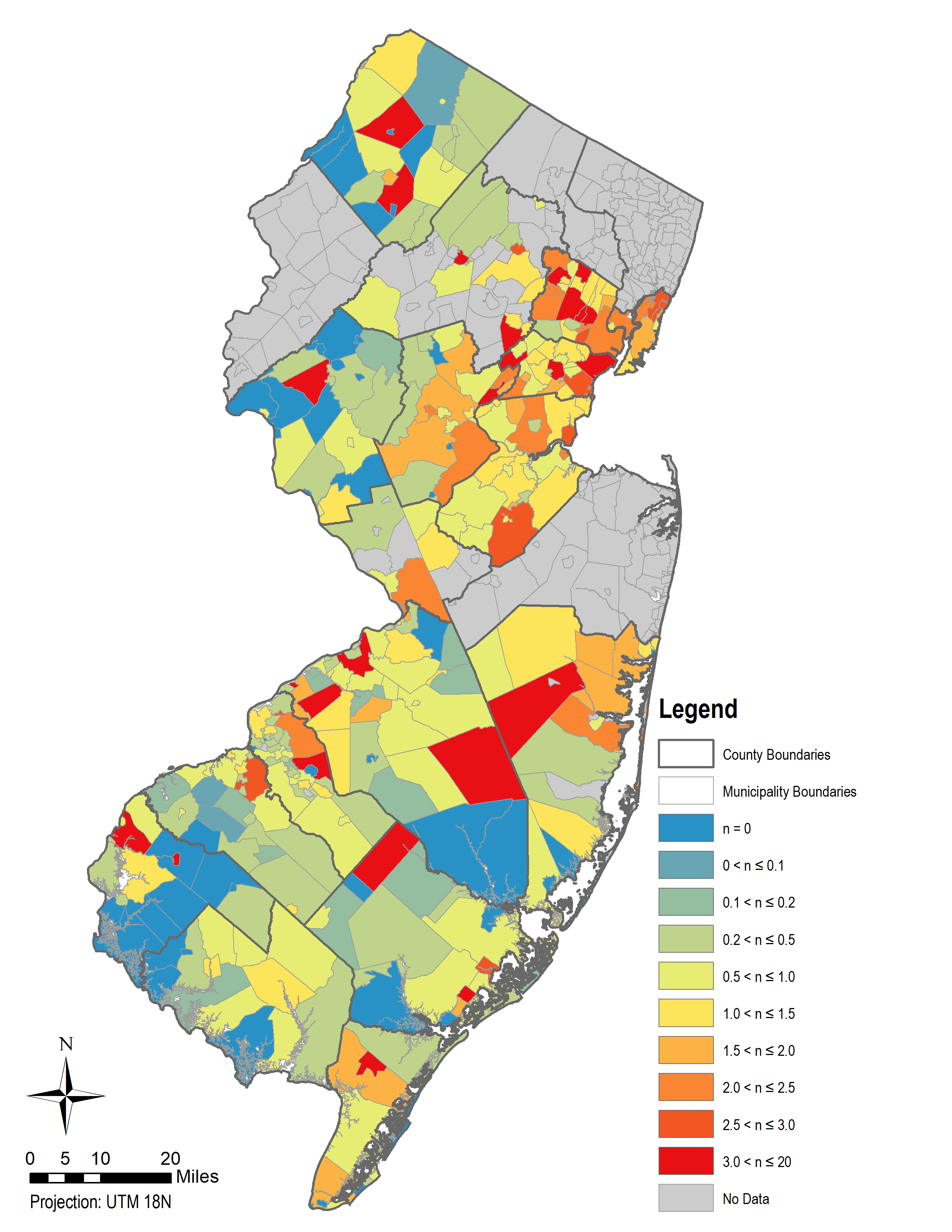

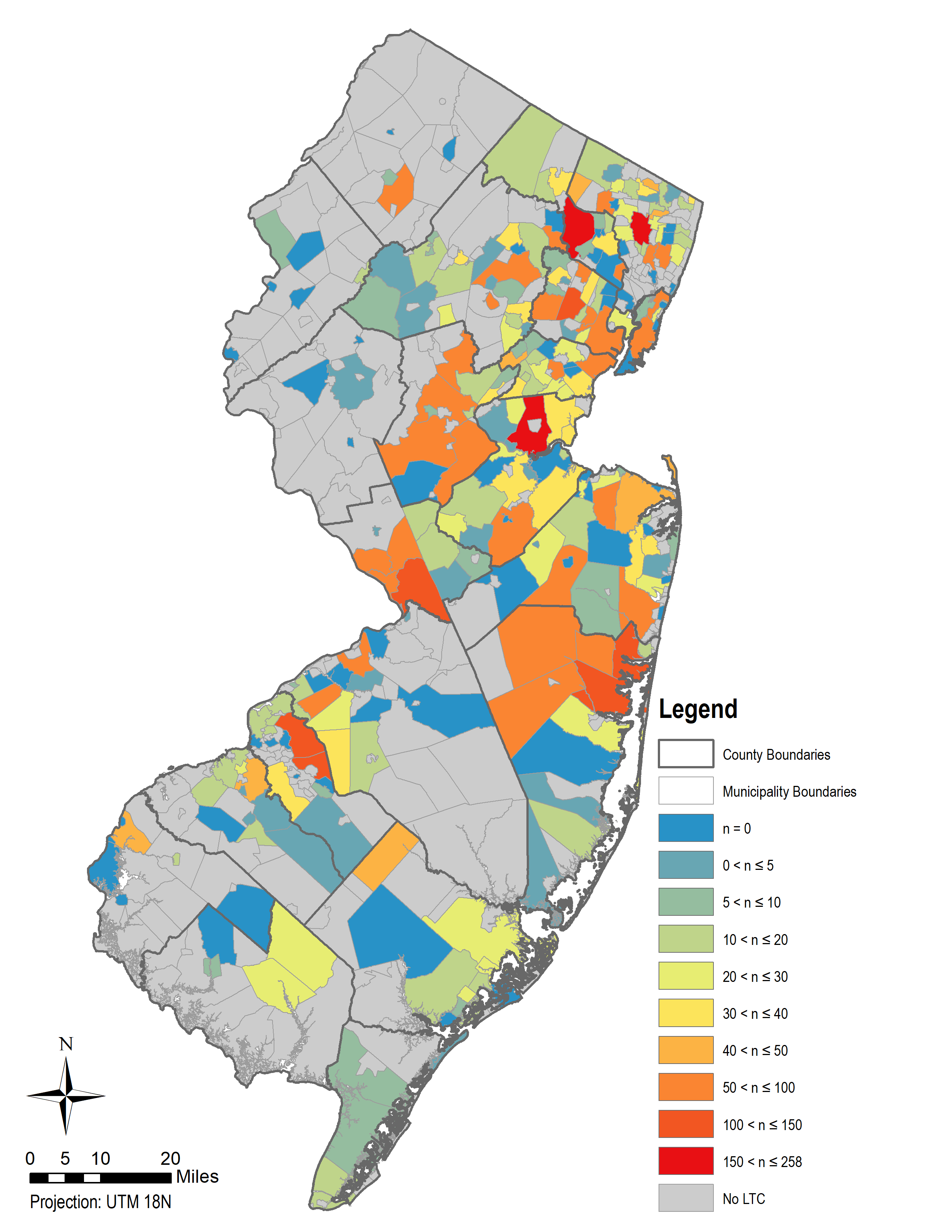

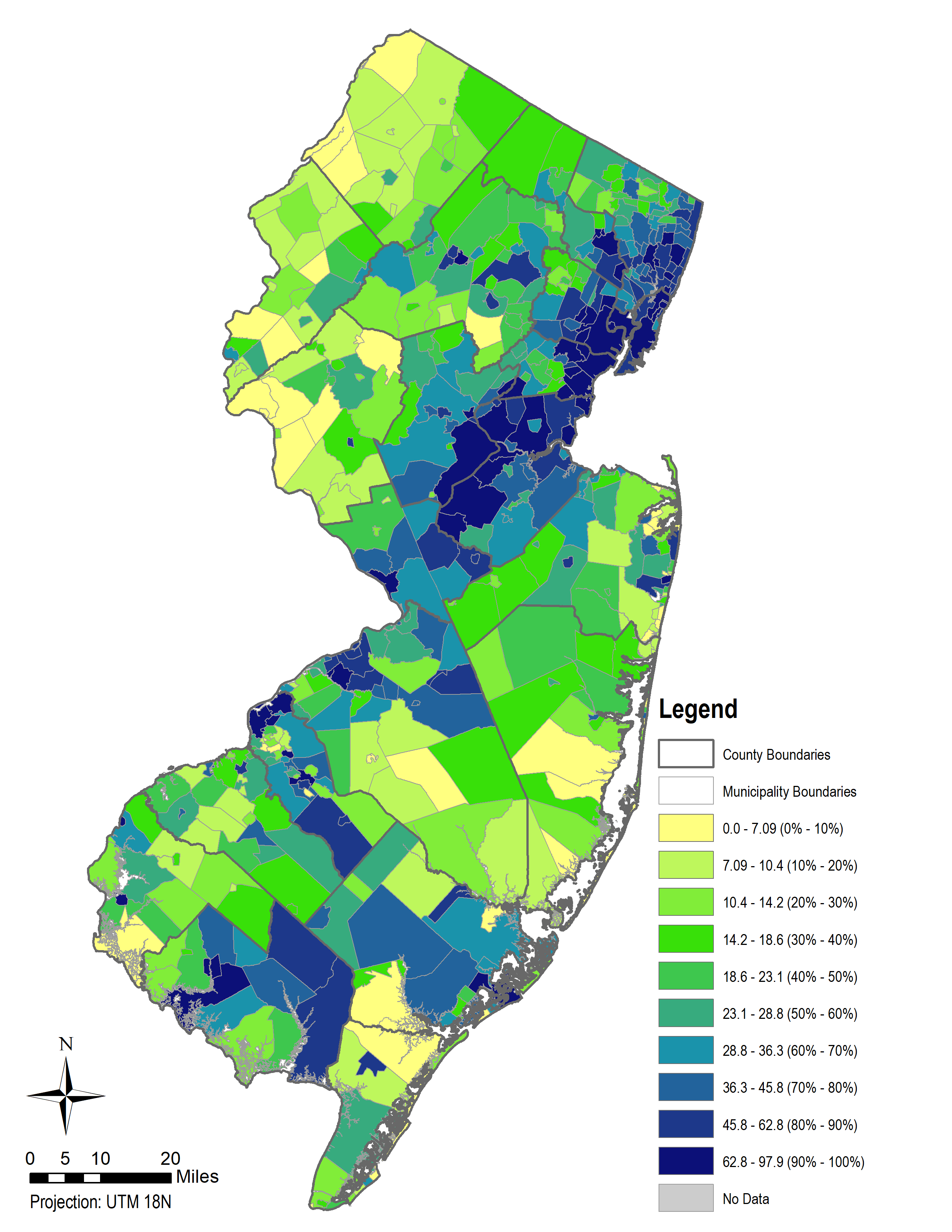

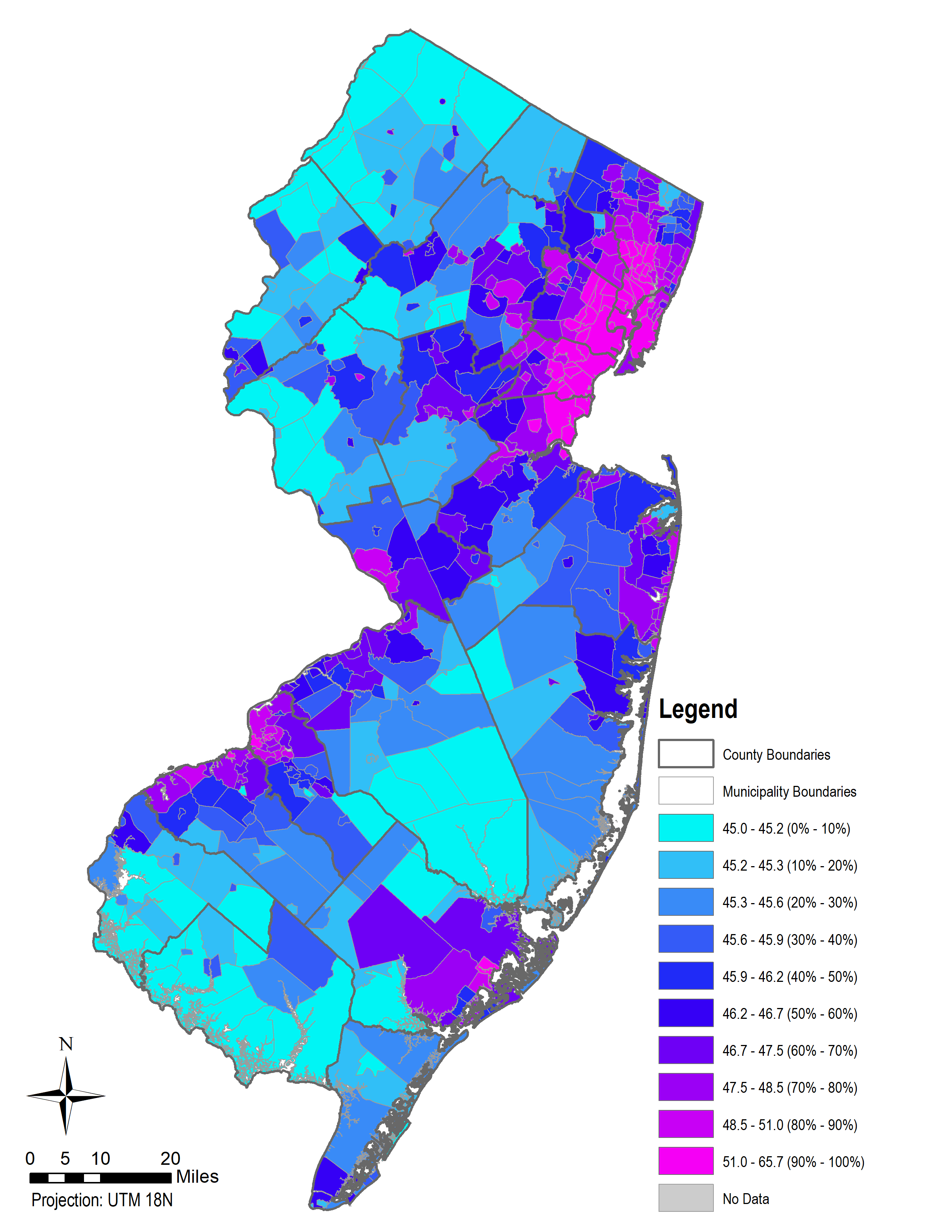

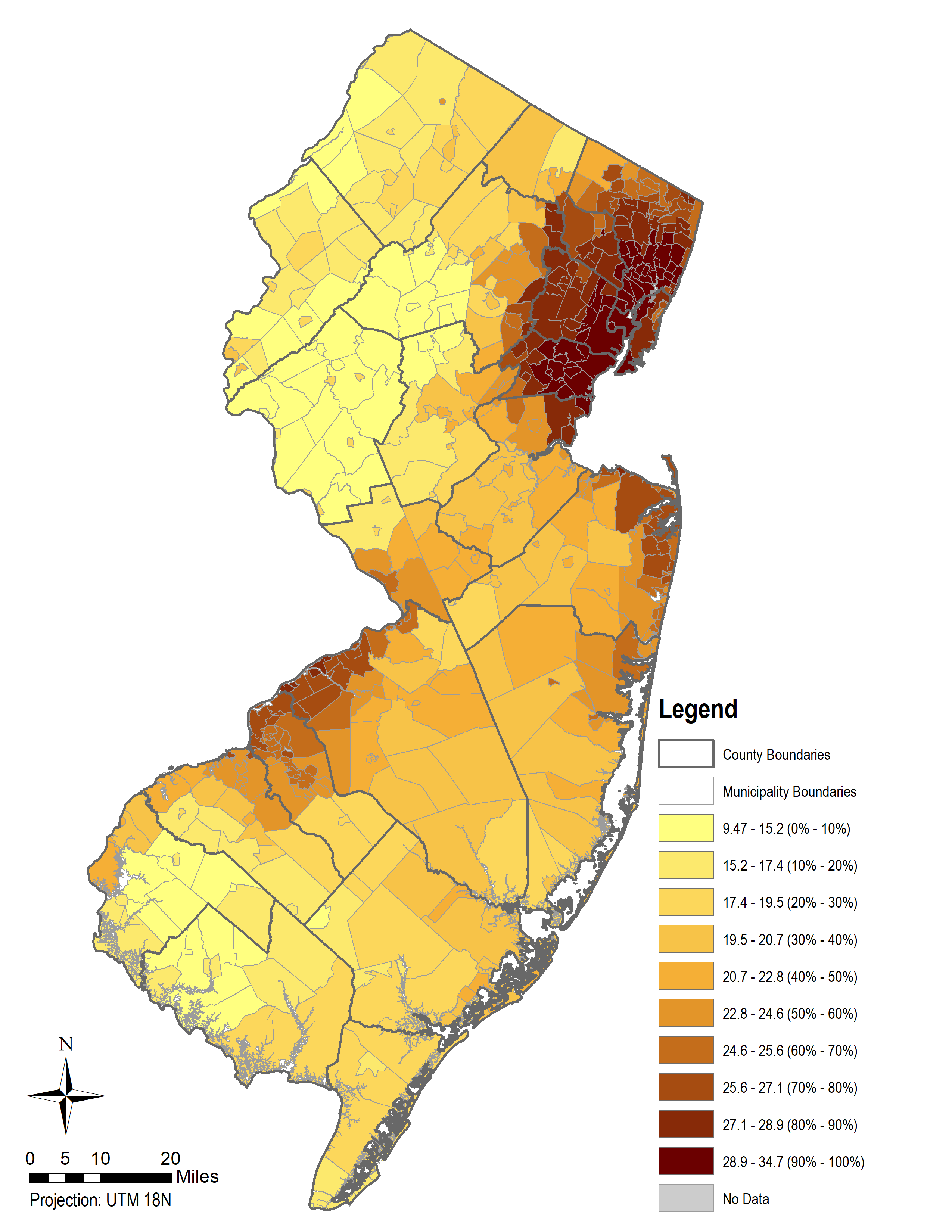


**DOT Noise Level**

**NO_2_ Average Concentration**

**% Minority**

**COVID-19 Death Rates**

**# COVID-19 Deaths (LTCF)**

**COVID-19 Case Rates**

**Fig. S2** New Jersey COVID-19 adverse health outcomes (at the end of the first phase of the pandemic, September 24, 2020), and socioexposomic factors by municipality.


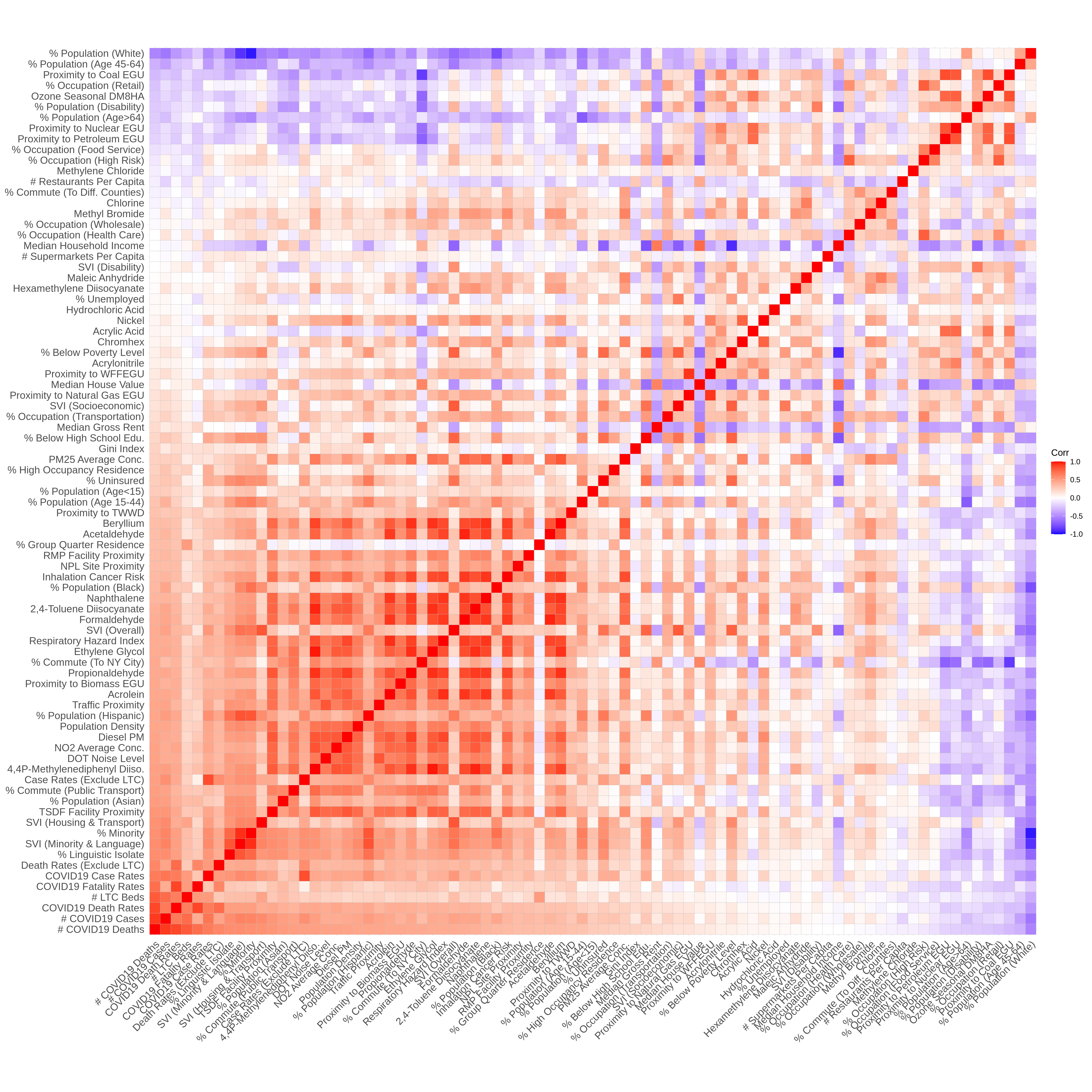


**Fig. S3** Correlations of the numbers of confirmed COVID-19 cases and deaths with 76 socioexposomic factors for the 565 municipalities of New Jersey. The Spearman correlation matrix has been sorted in decreasing order (from bottom to top).


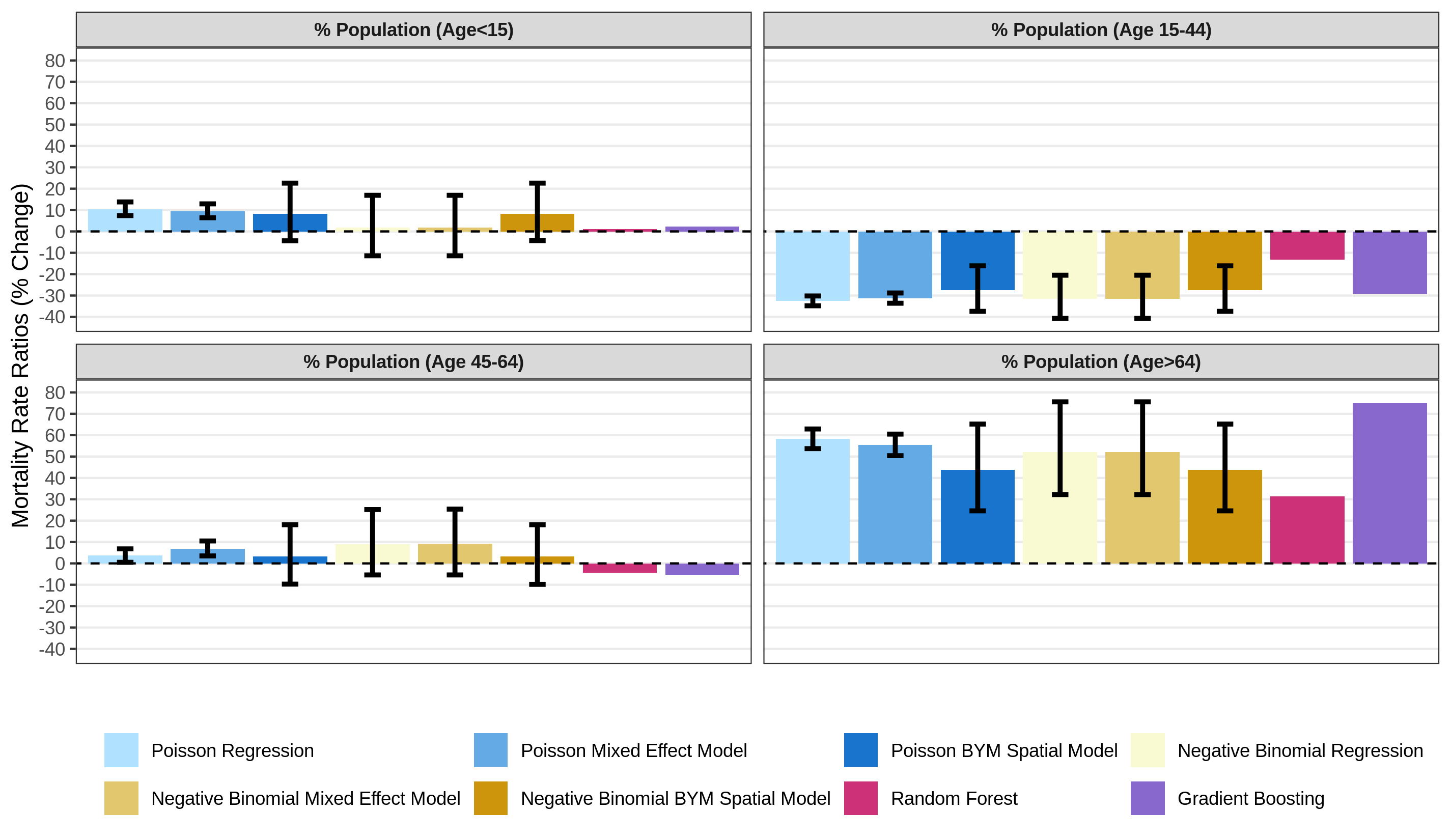


**Fig. S4**  Associations of municipality COVID-19 mortality rates with 4 factors representing different age groups, calculated from 8 geostatistical and machine learning models.


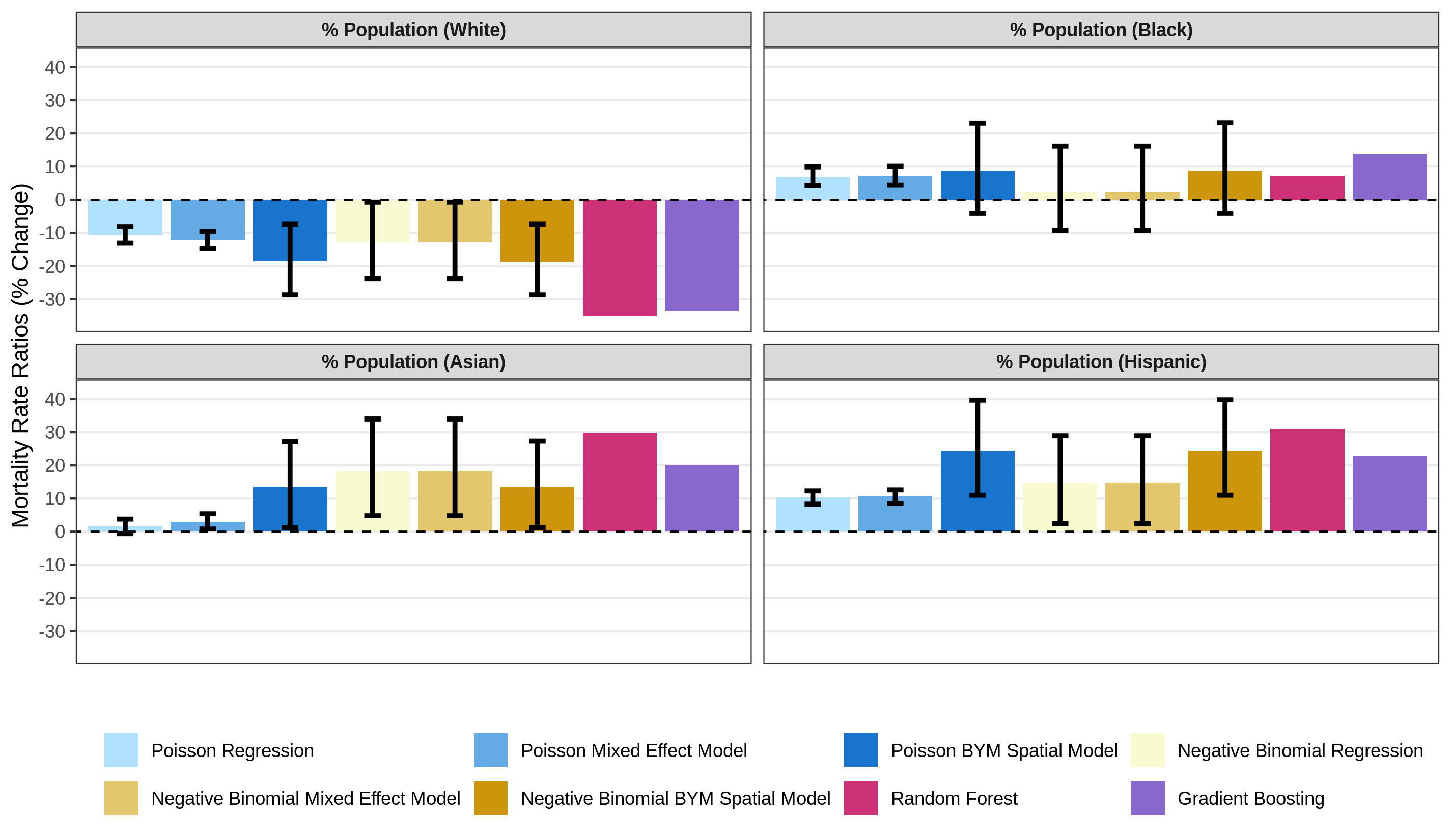


**Fig. S5** Associations of municipality COVID-19 mortality rates with 4 factors representing different racial and ethnic communities, calculated from 8 geostatistical and machine learning models.


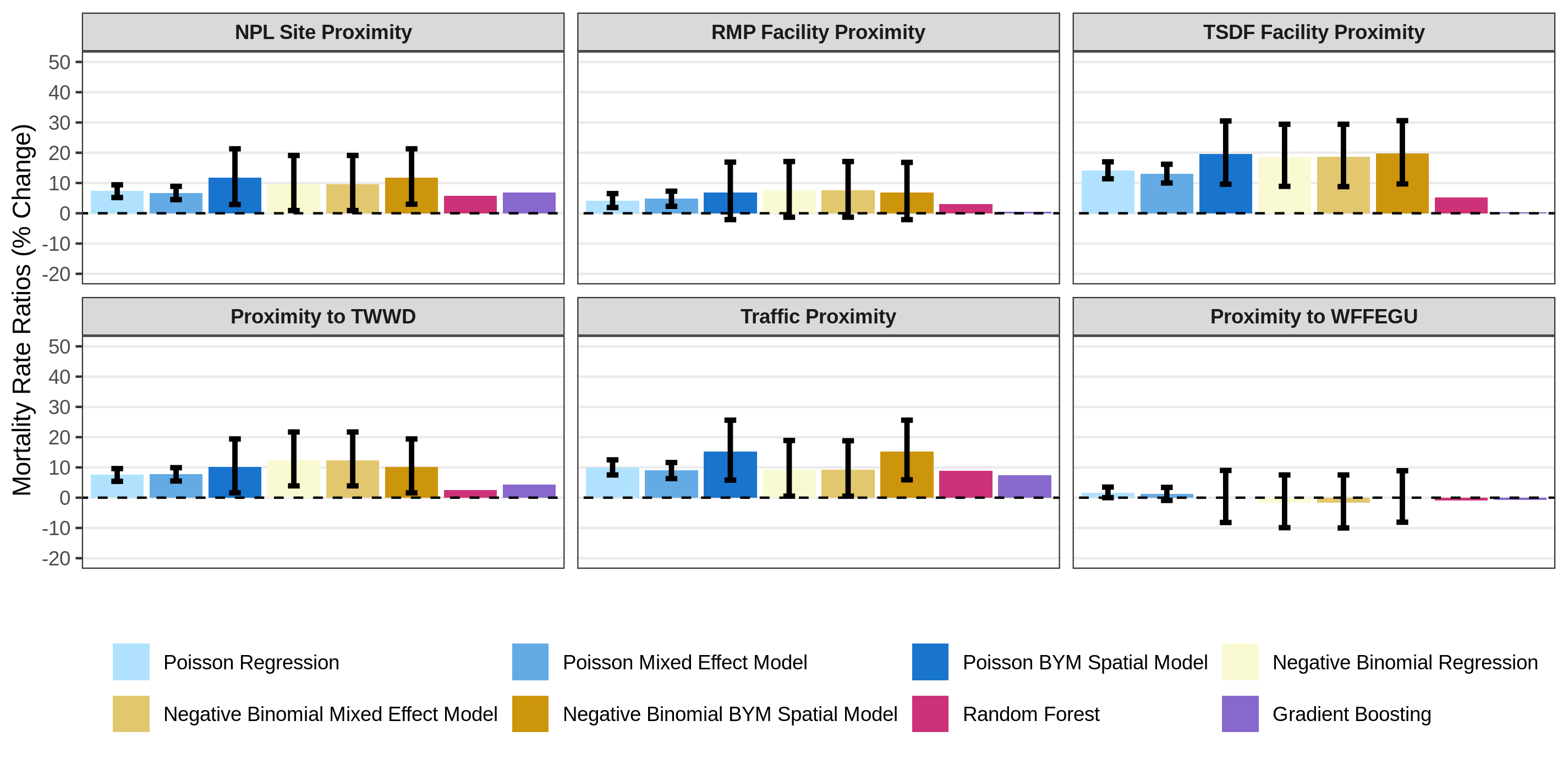


**Fig. S6**  Associations of municipality COVID-19 mortality rates with 6 factors representing proximity to industrial sites/facilities, calculated from 8 geostatistical and machine learning models.


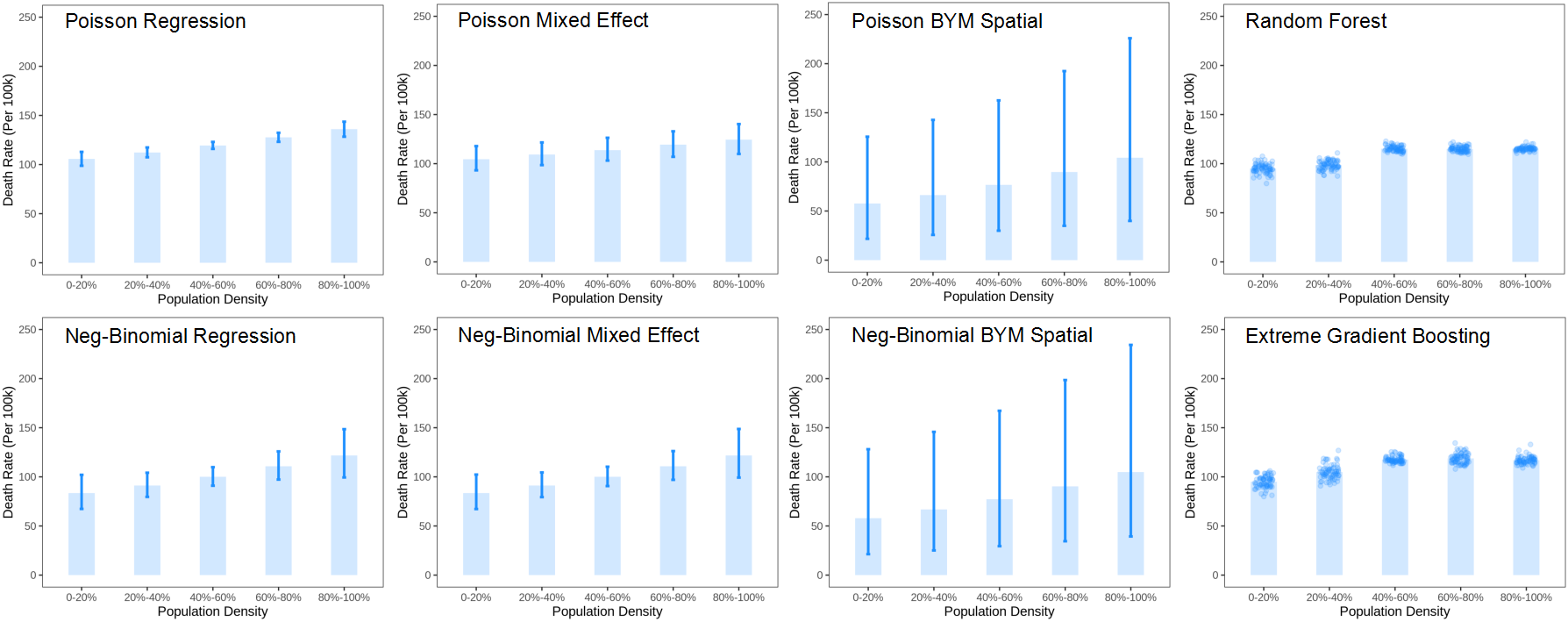


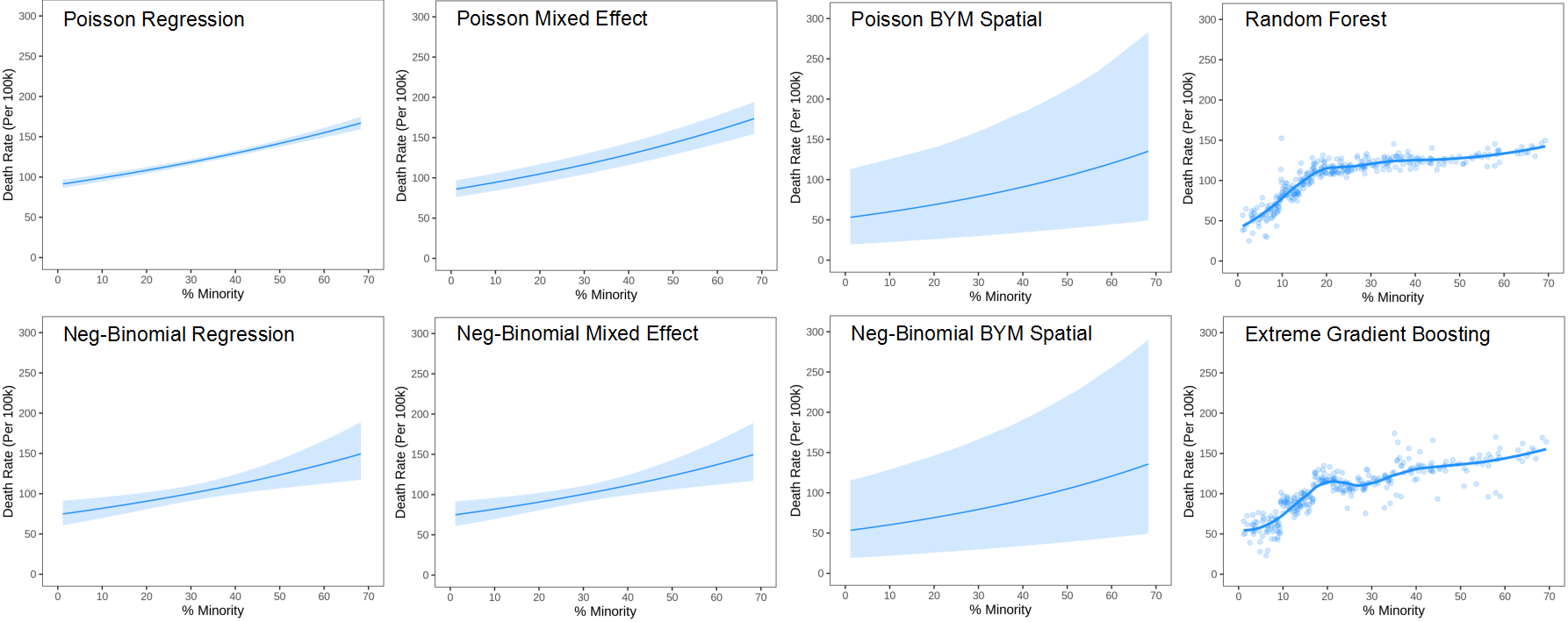


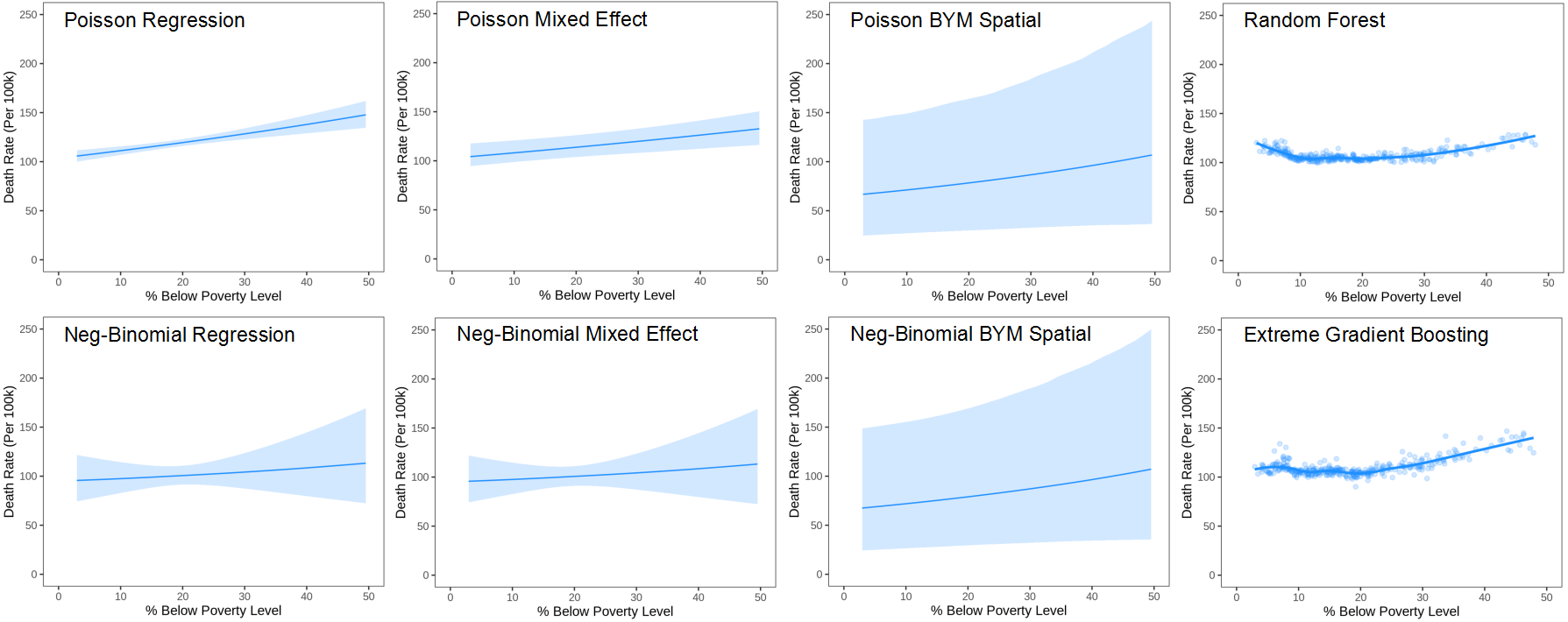


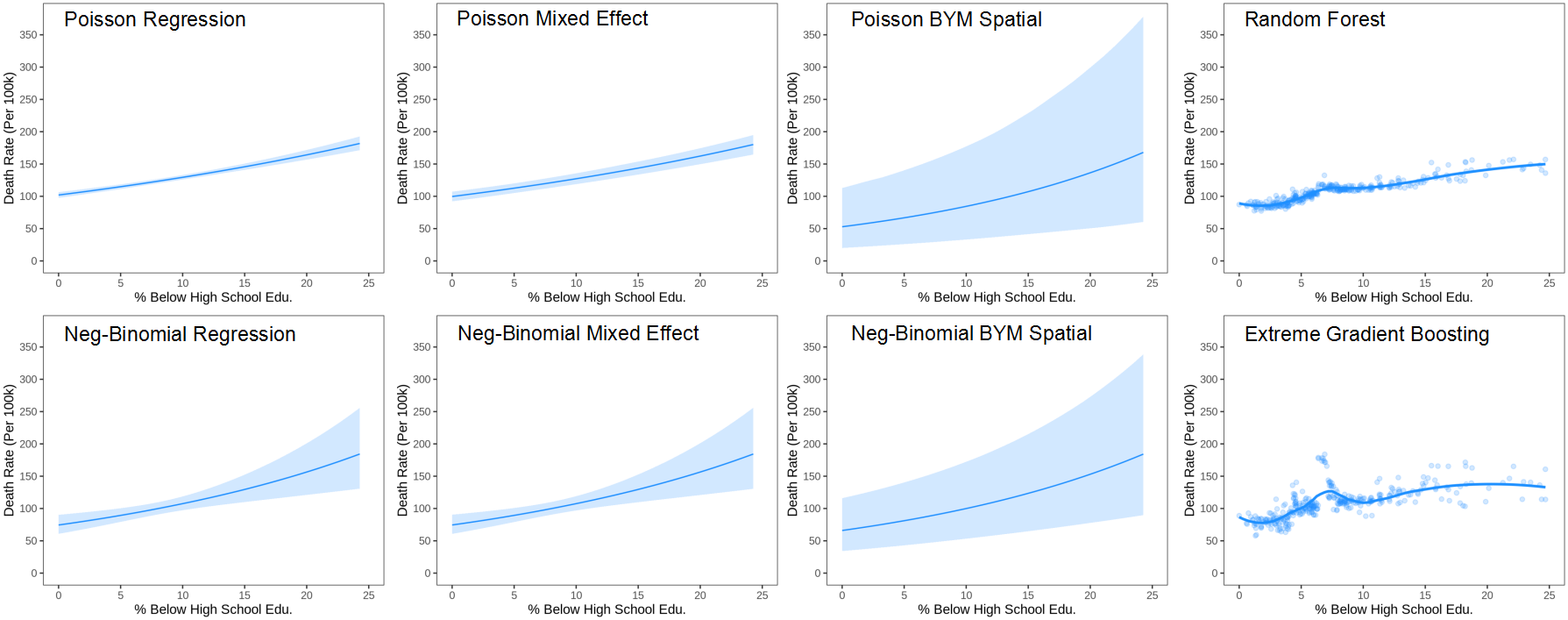


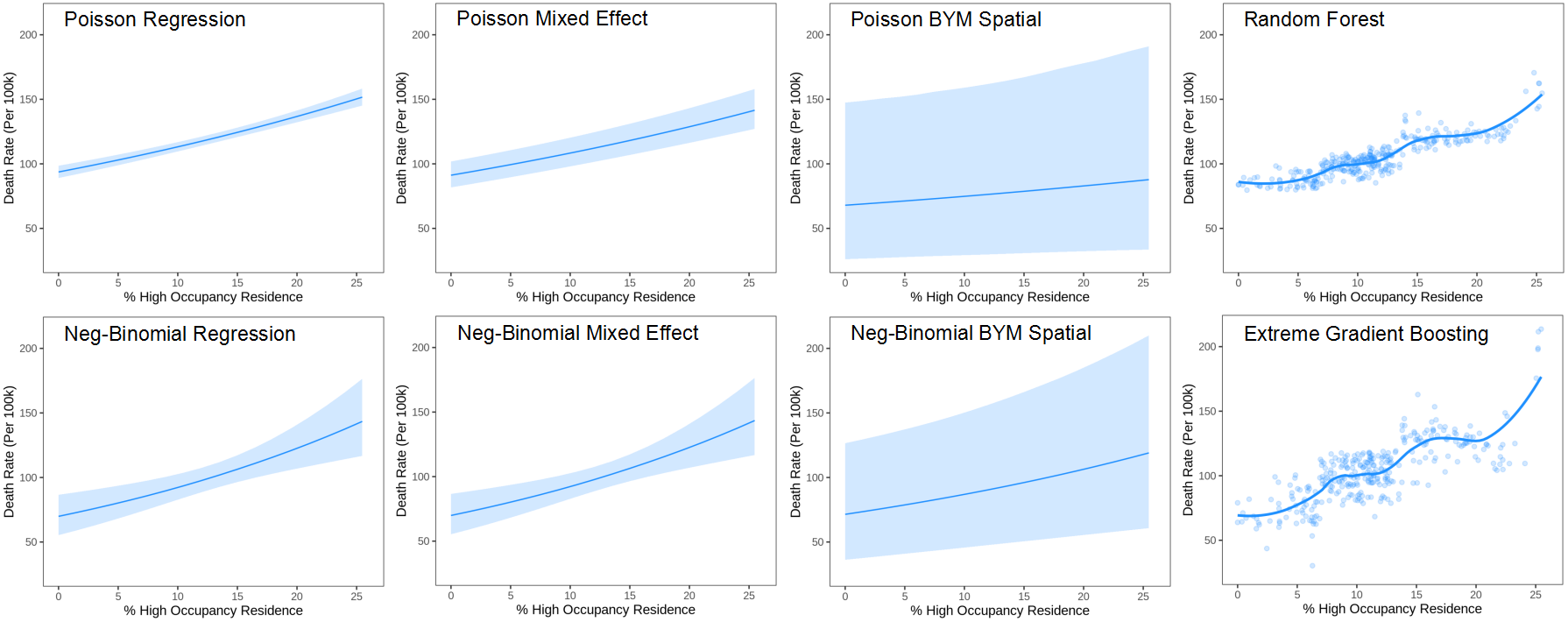


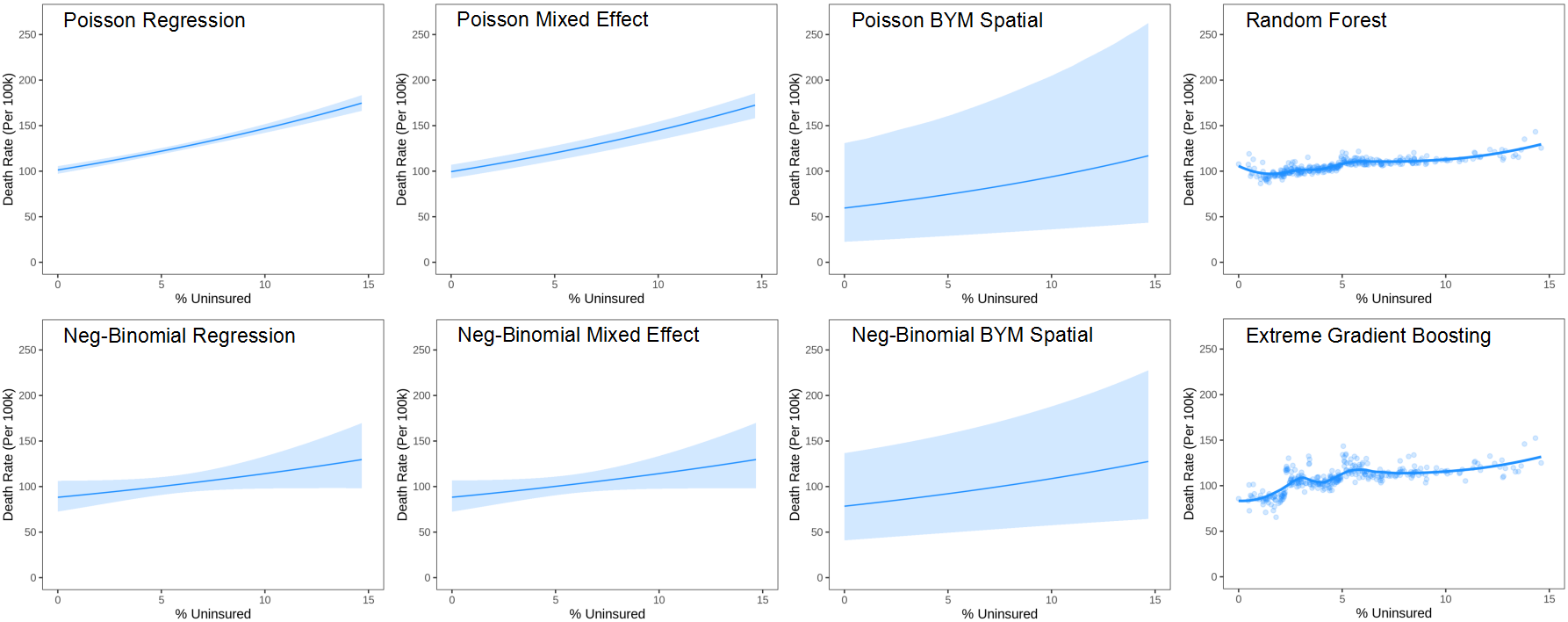


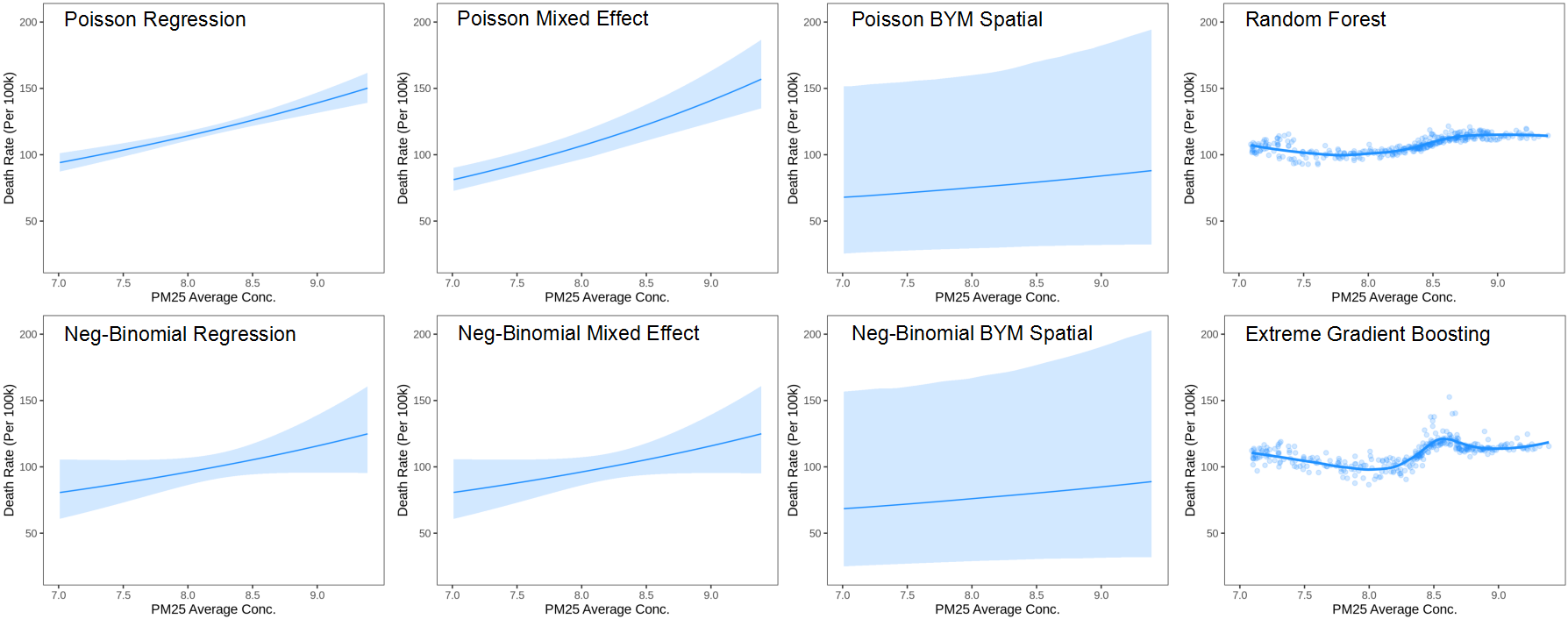


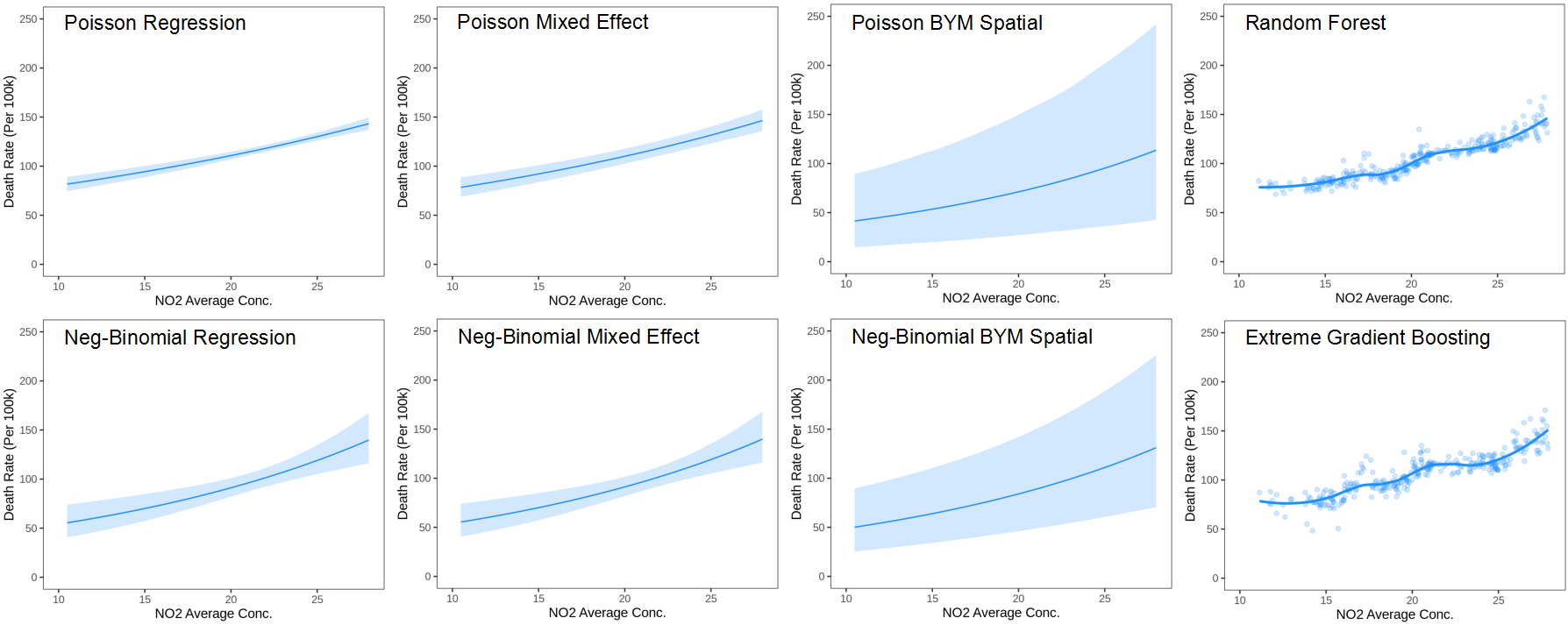


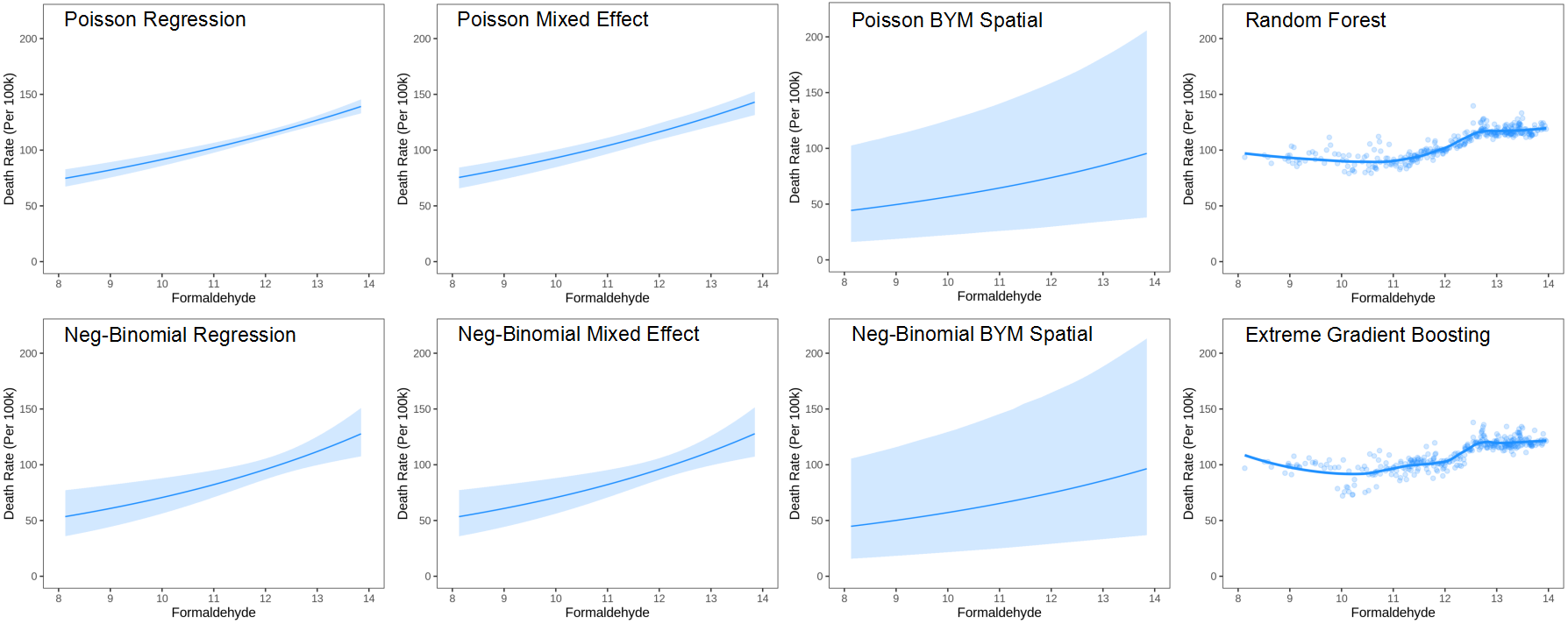


**Fig. S7**  Effects plots of 9 selected socioexposomic factors from 8 geostatistical and machine learning models.


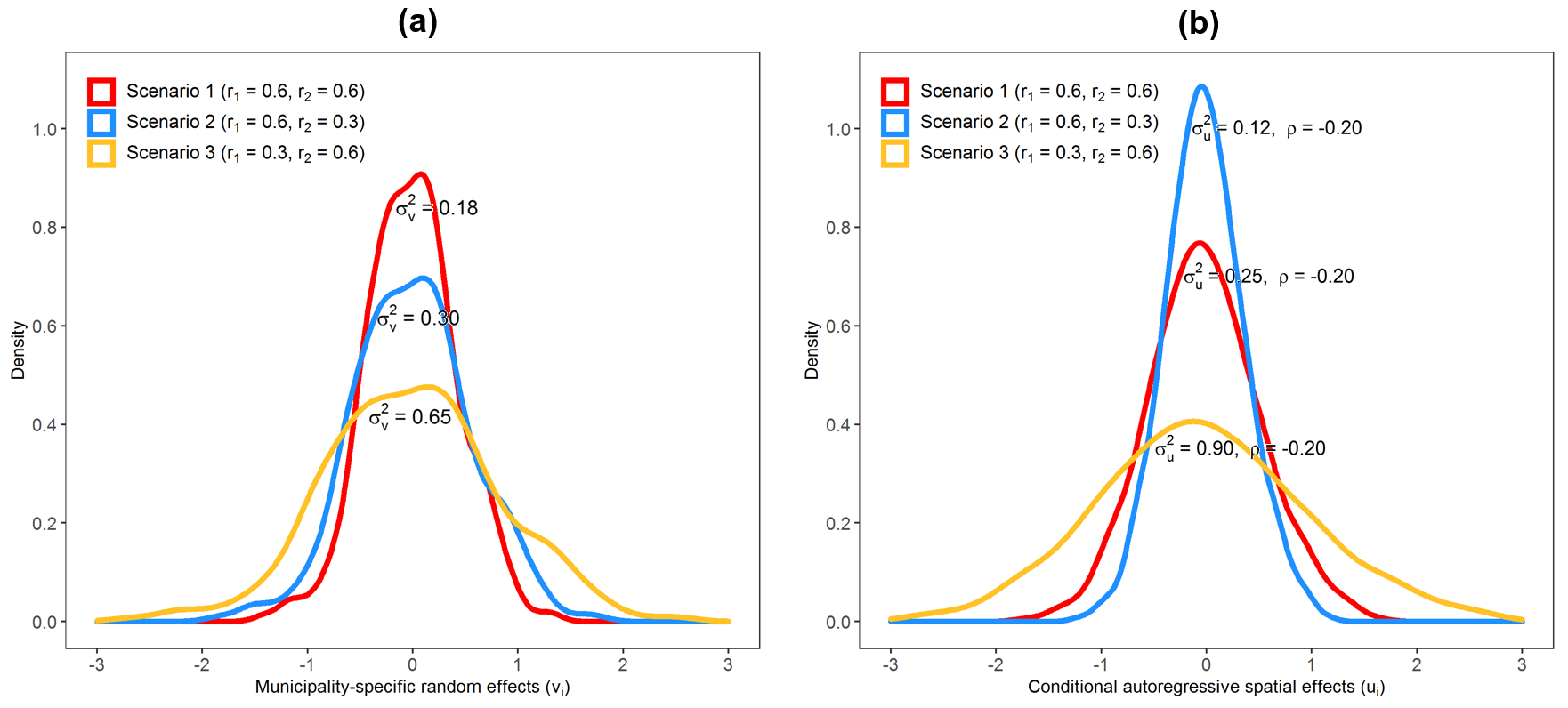


**Fig. S8** Simulation study: density plots of 565 samples drawing from (a) the ordinary (municipality-specific) random effect distribution and (b) the spatial (conditional autoregressive structural) random effect distribution, for each of the three scenarios.


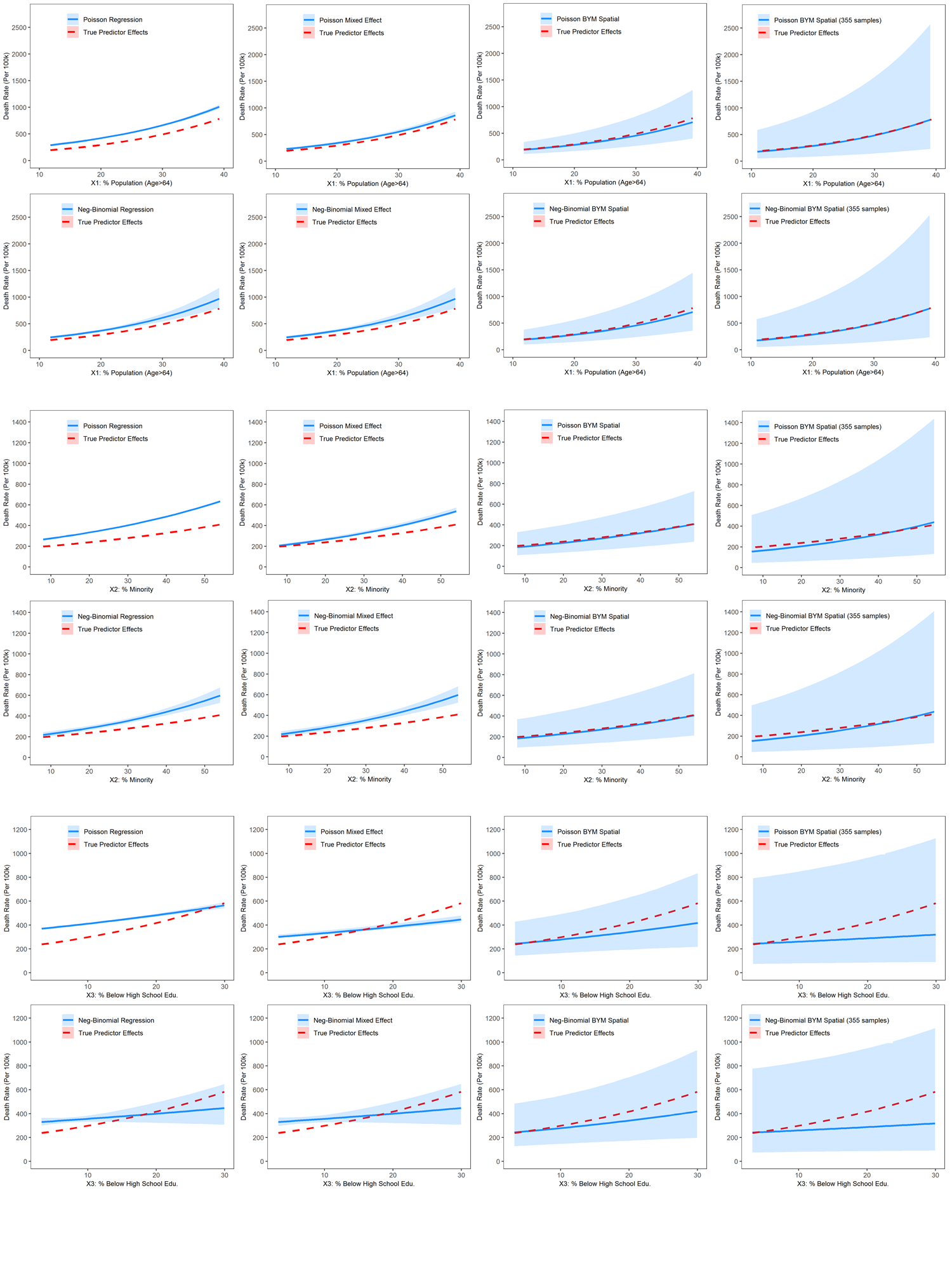


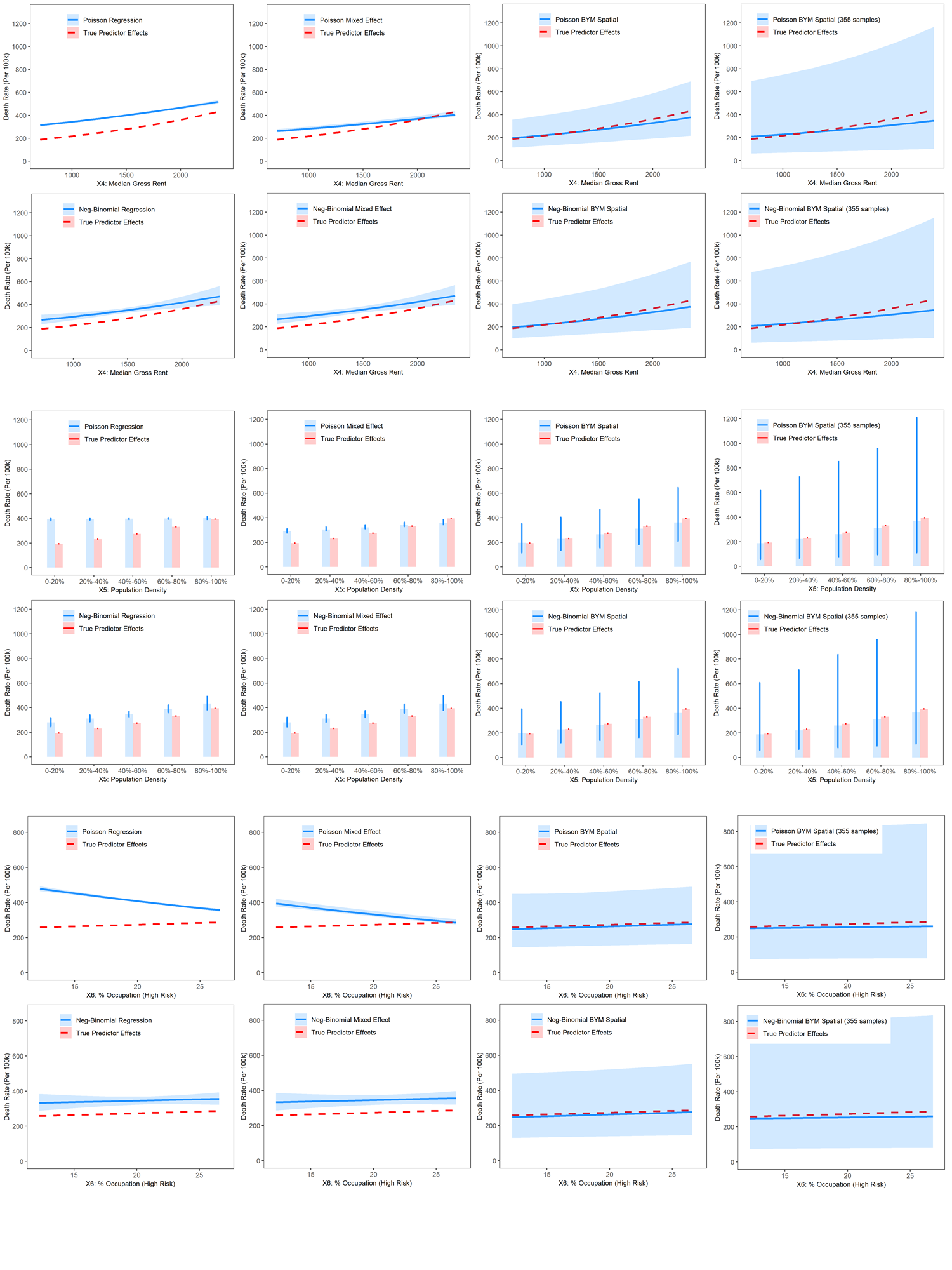


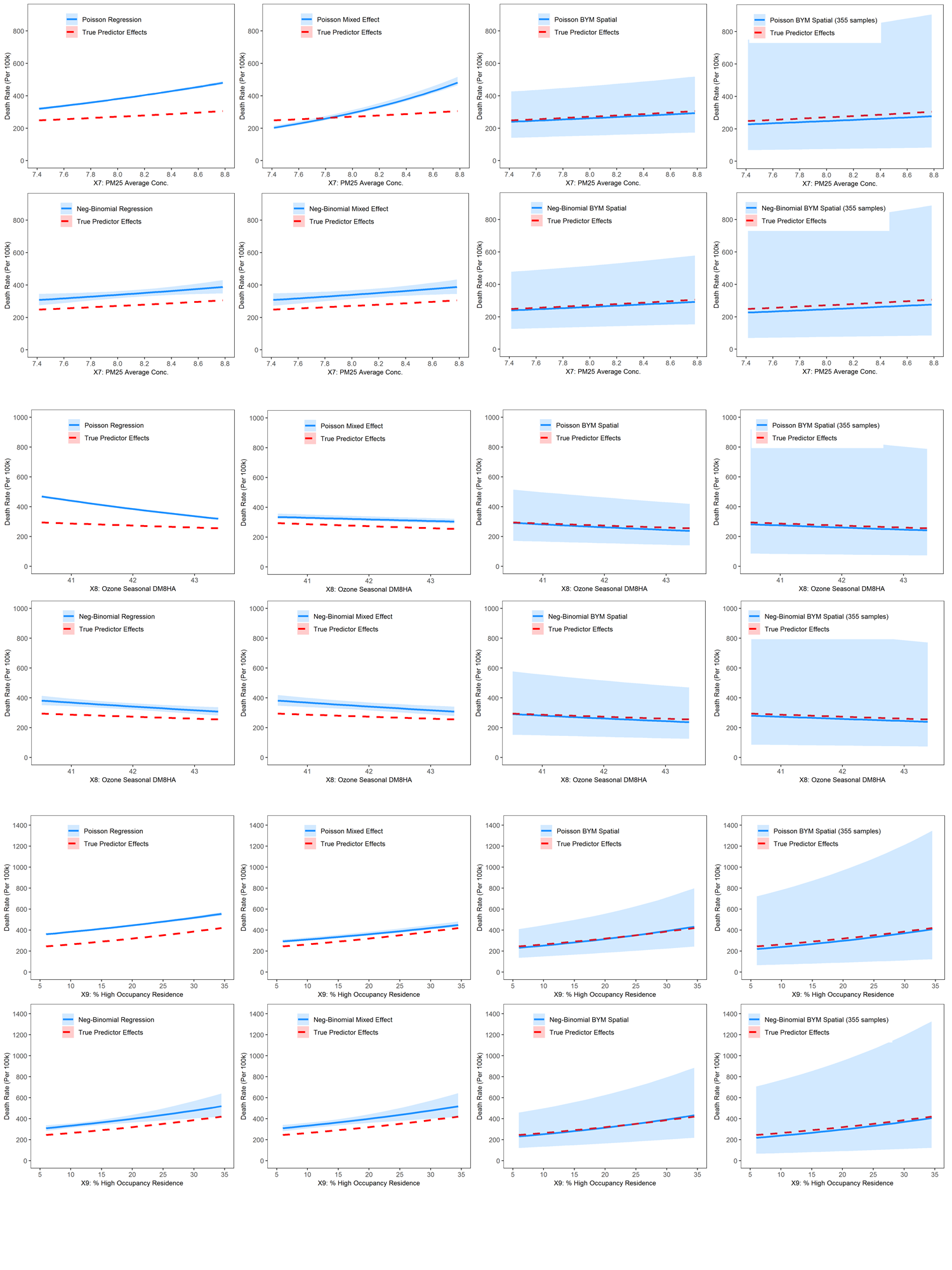


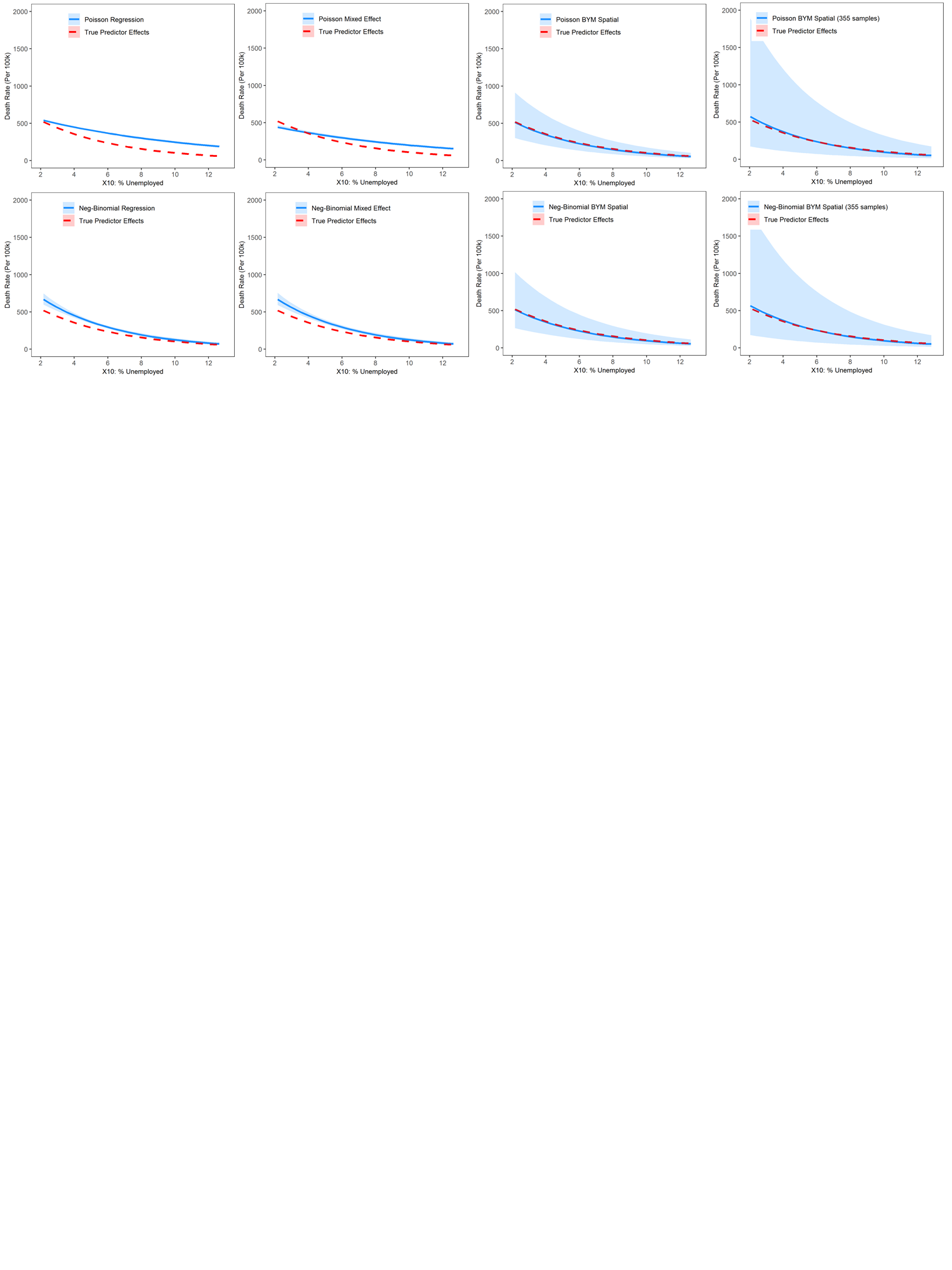


**Fig. S9** Simulation study: true and estimated predictor effects profiles of 10 variables from 8 statistical and geospatial models.

**
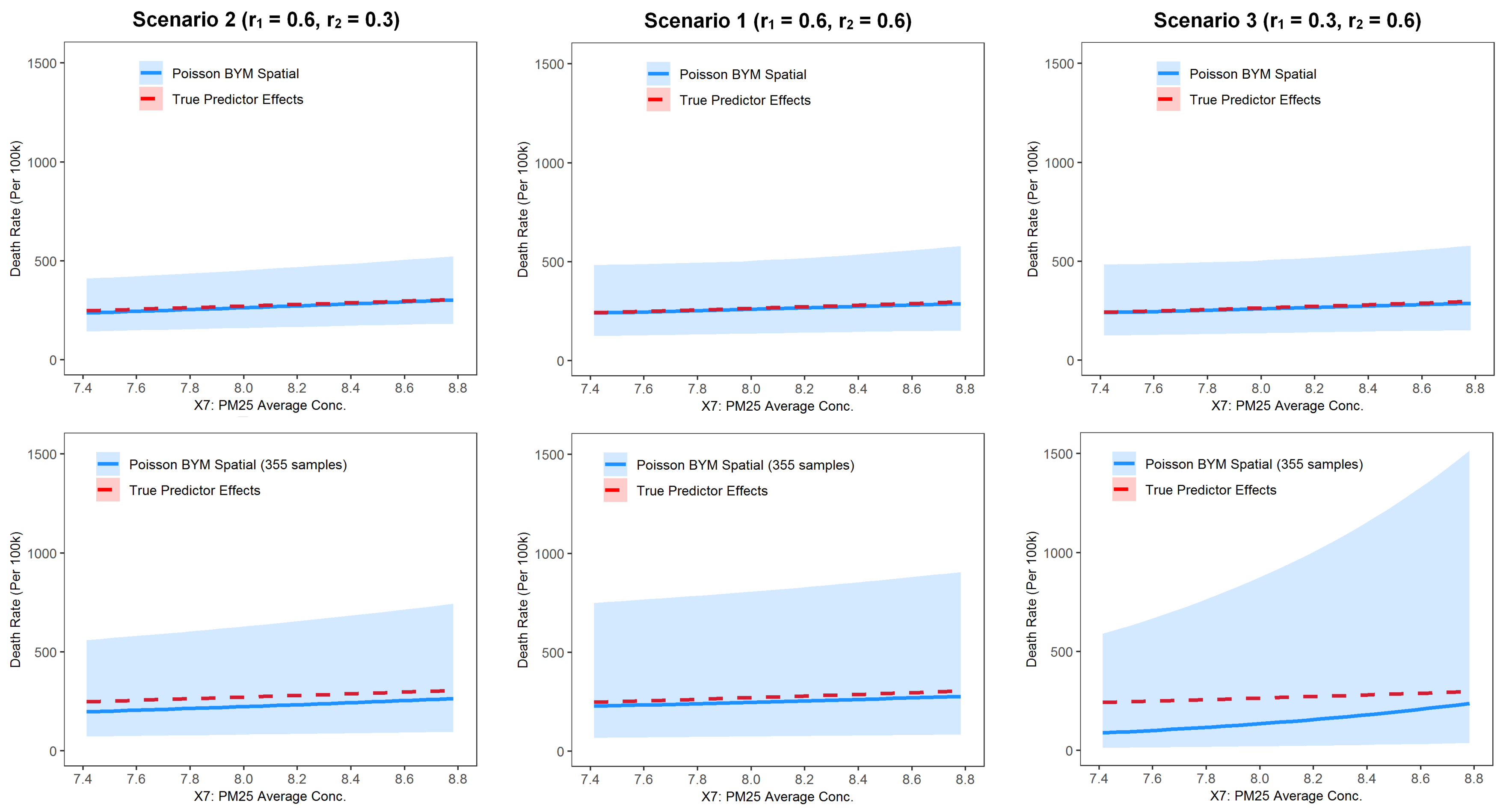
**

**Fig. S10** Simulation study: true and estimated predictor effects profiles of the selected variable (X7) from Poisson BYM spatial models for three scenarios. The first row corresponds to models fitted to 565 samples, and the second row corresponds to models fitted to 356 samples.


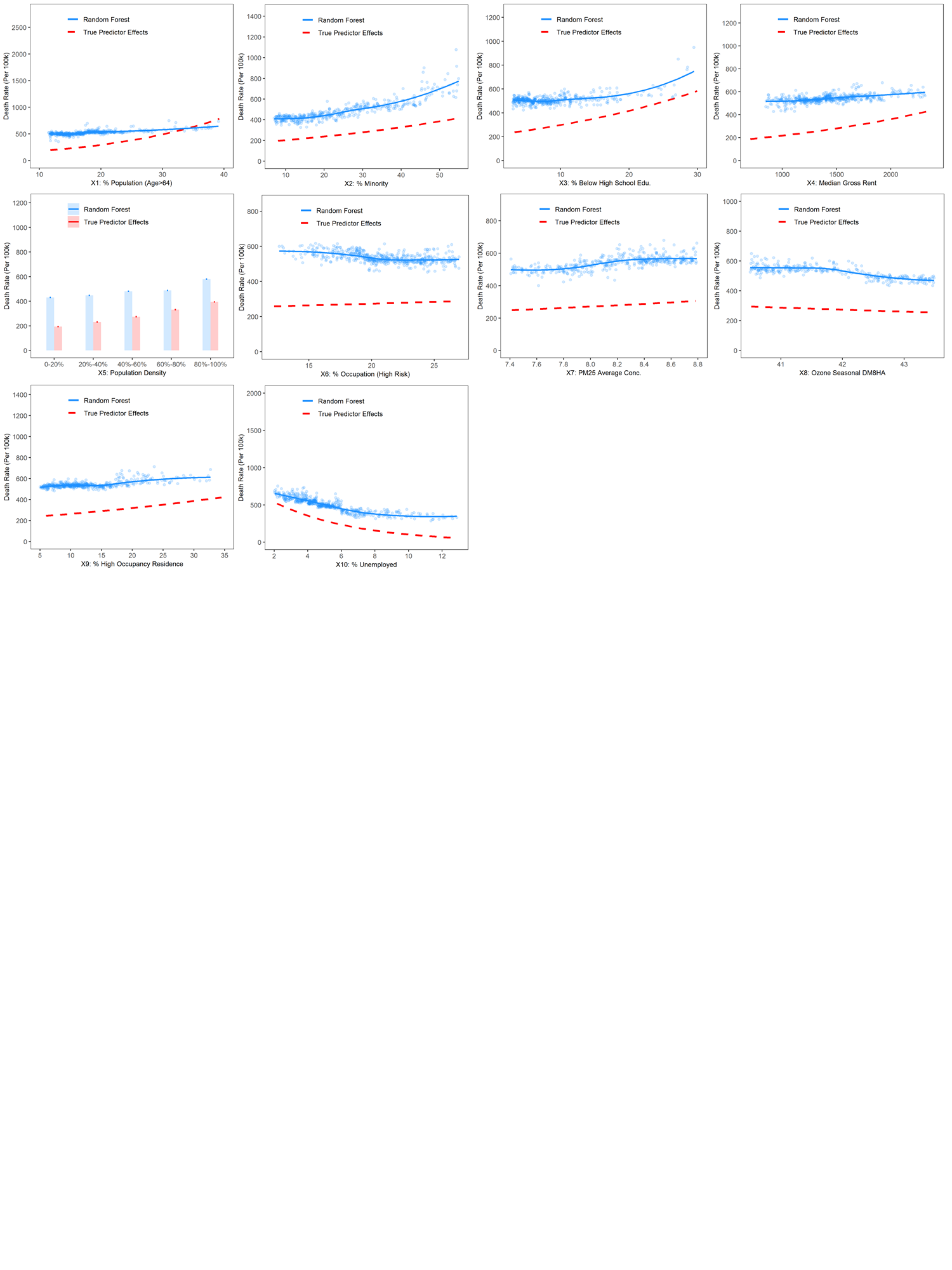


**Fig. S11** Simulation study: true predictor effects profiles and estimated Shapley effects profiles of 10 variables from Random Forest.

**Table S1. Description of multiple factors and adverse health outcomes for the COVID-19 socioexposomic New Jersey study. Factors comprising the socioexposome include air pollution, proximity to industrial sites, transportation-related noise, occupation and commuting, housing/neighborhood characteristics, among a wide range of environmental, demographic and socioeconomic metrics. These factors were aggregated at the municipality level through population weighted averaging.**

| **Data Category** | **Variable Name** | **Variable Description** | **Original Resolution** | **Year/Date** | **Data Source^c^** |
| --- | --- | --- | --- | --- | --- |
| **COVID-19 Cases/Deaths** | # COVID-19 Deaths | Number of reported COVID-19 deaths | Municipality | 9/24/2020 | Local health departments |
|  | COVID-19 Death Rates | Number of reported COVID-19 deaths per 1000 people | Municipality | 9/24/2020 | Local health departments |
|  | Death Rates (Exclude LTC^a^) | Number of reported COVID-19 deaths (excluding deaths occurred in Long-Term-Care facilities) per 1000 people | Municipality | 9/24/2020 | Local health departments |
|  | # COVID-19 Cases | Number of reported COVID-19 cases | Municipality | 9/24/2020 | Local health departments |
|  | COVID-19 Case Rates | Number of reported COVID-19 cases per 1000 people | Municipality | 9/24/2020 | Local health departments |
|  | Case Rates (Exclude LTC) | Number of reported COVID-19 cases (excluding cases occurred in Long-Term-Care facilities) per 1000 people | Municipality | 9/24/2020 | Local health departments |
|  | COVID-19 Fatality Rates | Number of reported COVID-19 deaths per 1000 cases | Municipality | 9/24/2020 | Local health departments |
| **Demographic Characteristics** | Population Density | Population per square mile | Municipality | 2015-2019 | ACS |
|  | % Population (Age < 15) | Percentage of population younger than 15 years old | Municipality | 2015-2019 | ACS |
|  | % Population (Age 15-44) | Percentage of population between age 15 and 44 years old | Municipality | 2015-2019 | ACS |
|  | % Population (Age 45-64) | Percentage of population between age 45 and 64 years old | Municipality | 2015-2019 | ACS |
|  | % Population (Age > 64) | Percentage of population older than 64 years old | Municipality | 2015-2019 | ACS |
|  | % Population (White) | Percentage of population, White alone | Municipality | 2015-2019 | ACS |
|  | % Population (Black) | Percentage of population, Black alone | Municipality | 2015-2019 | ACS |
|  | % Population (Asian) | Percentage of population, Asian alone | Municipality | 2015-2019 | ACS |
|  | % Population (Hispanic) | Percentage of population, Hispanic | Municipality | 2015-2019 | ACS |
|  | % Minority | Percentage of total minority population | Municipality | 2015-2019 | ACS |
| **Socioeconomic Status** | % Below High School Edu. | Percentage of population with less than high school diploma | Municipality | 2015-2019 | ACS |
|  | % Linguistic Isolate | Percentage of language isolated household | Municipality | 2015-2019 | ACS |
|  | % Below Poverty Level | Percentage of population living below 2x poverty level | Municipality | 2015-2019 | ACS |
|  | Gini Index | Gini index | Municipality | 2015-2019 | ACS |
|  | Median Gross Rent | Median gross rent | Municipality | 2015-2019 | ACS |
|  | Median Household Income | Median household income | Municipality | 2015-2019 | ACS |
|  | Median House Value | Median house value | Municipality | 2015-2019 | ACS |
|  | % High Occupancy Residence | Percentage of population living in household with more than 5 people or group quarter | Municipality | 2015-2019 | ACS |
|  | % Group Quarter Residence | Percentage of population live in group quarter | Municipality | 2015-2019 | ACS |
|  | % Population (Disability) | Percentage of population with disability of any type | Municipality | 2015-2019 | ACS |
|  | % Uninsured | Percentage of population with no health insurance | Municipality | 2015-2019 | ACS |
|  | % Unemployed | Percentage of unemployed people (over 16 years old in labor force without a job divided by total population) | Municipality | 2015-2019 | ACS |
|  | SVI^b^ (Socioeconomic) | Social Vulnerability Index – Socioeconomic Status | Municipality | 2015-2019 | ACS |
|  | SVI (Disability) | Social Vulnerability Index - Household Composition & Disability | Municipality | 2015-2019 | ACS |
|  | SVI (Minority & Language) | Social Vulnerability Index - Minority Status & Language | Municipality | 2015-2019 | ACS |
|  | SVI (Housing & Transport) | Social Vulnerability Index - Housing Type & Transportation | Municipality | 2015-2019 | ACS |
|  | SVI (Overall) | Social Vulnerability Index - The overall Vulnerability Index | Municipality | 2015-2019 | ACS |
| **Air Pollutants** | PM25 Average Conc. | Modeled annual average daily mean ambient PM_2.5_ concentrations (μg/m^3^) | Block Group | 2016 | EJScreen |
|  | NO2 Average Conc. | Modeled annual average daily max 1 hour average ambient NO_2_ concentrations (ppb) | 1km x 1km | 2016 | Di et al. |
|  | Ozone Seasonal DM8HA | Modeled summer seasonal daily max 8 hour average ambient ozone concentration (ppb) | Block Group | 2016 | EJScreen |
|  | Acrolein | Modeled annual average hazard quotient of ambient Acrolein | Census Tract | 2014 | NATA |
|  | Acetaldehyde | Modeled annual average hazard quotient of ambient Acetaldehyde | Census Tract | 2014 | NATA |
|  | Formaldehyde | Modeled annual average hazard quotient of ambient Formaldehyde | Census Tract | 2014 | NATA |
|  | Diesel PM | Modeled annual average hazard quotient of ambient Diesel Particulate Matter | Census Tract | 2014 | NATA |
|  | Naphthalene | Modeled annual average hazard quotient of ambient Naphthalene | Census Tract | 2014 | NATA |
|  | Acrylic Acid | Modeled annual average hazard quotient of ambient Acrylic Acid | Census Tract | 2014 | NATA |
|  | Acrylonitrile | Modeled annual average hazard quotient of ambient Acrylonitrile | Census Tract | 2014 | NATA |
|  | Beryllium | Modeled annual average hazard quotient of ambient Beryllium | Census Tract | 2014 | NATA |
|  | Chlorine | Modeled annual average hazard quotient of ambient Chlorine | Census Tract | 2014 | NATA |
|  | Chromhex | Modeled annual average hazard quotient of ambient Chromhex | Census Tract | 2014 | NATA |
|  | Ethylene Glycol | Modeled annual average hazard quotient of ambient Ethylene Glycol | Census Tract | 2014 | NATA |
|  | Hexamethylene Diisocyanate | Modeled annual average hazard quotient of ambient Hexamethylene Diisocyanate | Census Tract | 2014 | NATA |
|  | Hydrochloric Acid | Modeled annual average hazard quotient of ambient Hydrochloric Acid | Census Tract | 2014 | NATA |
|  | Maleic Anhydride | Modeled annual average hazard quotient of ambient Maleic Anhydride | Census Tract | 2014 | NATA |
|  | Methylene Chloride | Modeled annual average hazard quotient of ambient Methylene Chloride | Census Tract | 2014 | NATA |
|  | Methyl Bromide | Modeled annual average hazard quotient of ambient Methyl Bromide | Census Tract | 2014 | NATA |
|  | Nickel | Modeled annual average hazard quotient of ambient Nickel | Census Tract | 2014 | NATA |
|  | Propionaldehyde | Modeled annual average hazard quotient of ambient Propionaldehyde | Census Tract | 2014 | NATA |
|  | 2,4-Toluene Diisocyanate | Modeled annual average hazard quotient of ambient 2,4-Toluene Diisocyanate | Census Tract | 2014 | NATA |
|  | 4,4P-Methylenediphenyl Diiso. | Modeled annual average hazard quotient of ambient 4,4P-Methylenediphenyl Diiso. | Census Tract | 2014 | NATA |
|  | Inhalation Cancer Risk | Modeled Inhalation Cancer Risk | Census Tract | 2014 | NATA |
|  | Respiratory Hazard Index | Modeled Respiratory Hazard Index | Census Tract | 2014 | NATA |
| **Proximity to Industrial Sites** | NPL Site Proximity | Proximity to National Priorities List (NPL) sites | Block Group | 2019 | EJScreen |
|  | RMP Facility Proximity | Proximity to Risk Management Plan (RMP) facilities | Block Group | 2019 | EJScreen |
|  | TSDF Facility Proximity | Proximity to Treatment Storage and Disposal (TSDF) facilities | Block Group | 2019 | EJScreen |
|  | Proximity to TWWD | Proximity to toxicity-weighted wastewater discharges (TWWD) | Block Group | 2019 | EJScreen |
|  | Traffic Proximity | Traffic proximity and volume | Block Group | 2019 | EJScreen |
|  | Proximity to Natural Gas EGU | Proximity to natural gas power plant | Facility | 2020 | NJDEP |
|  | Proximity to Petroleum EGU | Proximity to petroleum power plant | Facility | 2020 | NJDEP |
|  | Proximity to Biomass EGU | Proximity to biomass power plant | Facility | 2020 | NJDEP |
|  | Proximity to Coal EGU | Proximity to coal Power Plant | Facility | 2020 | NJDEP |
|  | Proximity to Nuclear EGU | Proximity to nuclear power plant | Facility | 2020 | NJDEP |
|  | Proximity to WFFEGU | Proximity to all types of Fossil-Fuel based power plant | Facility | 2020 | NJDEP |
| **Transportation Related Noise** | DOT Noise Level | Modeled 24-Hr average transportation-related noise level (dB(A)) | 30m x 30m | 2018 | BTS |
| **Occupation and Commuting** | % Occupation (Wholesale) | Percentage of population having jobs in Wholesale Trade | Block | 2018 | LEHD |
|  | % Occupation (Retail) | Percentage of population having jobs in Retail Trade | Block | 2018 | LEHD |
|  | % Occupation (Transportation) | Percentage of population having jobs in Transportation and Warehousing | Block | 2018 | LEHD |
|  | % Occupation (Health Care) | Percentage of population having jobs in Health Care and Social Assistance | Block | 2018 | LEHD |
|  | % Occupation (Food Service) | Percentage of population having jobs in Accommodation and Food Services | Block | 2018 | LEHD |
|  | % Occupation (High Risk) | Percentage of population having jobs in high risk industries | Block | 2018 | LEHD |
|  | % Commute (To Diff. Counties) | Percentage of population having jobs in another county | Block | 2018 | LEHD |
|  | % Commute (To NY City) | Percentage of population having jobs in New York City | Block | 2018 | LEHD |
|  | % Commute (Public Transport) | Percentage of population using public transportation to work | Municipality | 2019 | ACS |
| **Neighborhood Facilities** | # LTC Beds | Number of licensed Long-Term-Care beds within the township (excluding pediatric day health care services) | Municipality | 2020 | NJDOH |
|  | # Restaurants Per Capita | Full-service restaurants per 1,000 people | zip code | 2018 | AHRQ |
|  | # Supermarkets Per Capita | Supermarkets and other grocery (except convenience) stores per 1,000 people | zip code | 2018 | AHRQ |

^a^Long-term-care deaths/cases were retrieved from NJDOH.

^b^SVI estimates were calculated using the 15 individual socioeconomic variables across all municipalities in New Jersey, applying the same methodology developed by CDC (<https://www.atsdr.cdc.gov/placeandhealth/svi/index.html>).

^c^Local health departments: <https://ccl-eohsi.shinyapps.io/covid19_dashboard/>

ACS (American Community Survey): <https://www.census.gov/data/developers/data-sets/acs-5year.html>

EJSCREEN (Environmental Justice Screening and Mapping Tool): <https://www.epa.gov/ejscreen/download-ejscreen-data>

NATA (National Air Toxics Assessment): <https://www.epa.gov/national-air-toxics-assessment>

NJDEP (New Jersey Department of Environmental Protection):

<https://njogis-newjersey.opendata.arcgis.com/datasets/njdep::power-plants-of-new-jersey/>

BTS (Bureau of Transportation Statistics): <https://maps.dot.gov/BTS/NationalTransportationNoiseMap/>

LEHD (Longitudinal Employer-Household Dynamics): [https://lehd.ces.census.gov/data/#lodes](https://lehd.ces.census.gov/data/#lodes)

AHRQ **(**Agency for Healthcare Research and Quality**):** <http://www.ahrq.gov/sdoh/data-analytics/sdoh-data.html>

**Table S2.** Structures and relevant descriptions for the six geostatistical models implemented in this study.

| **Model** | **Distribution** | **Parameter^g^** | **DIC^h^** |
| --- | --- | --- | --- |
| Poisson^a^ | Poisson | **β** | 4266.35 |
| Poisson Mixed^b^ | Poisson | **β**, *v_i_* | 4191.49 |
| Poisson BYM^c^ | Poisson | **β**, *v_i_, u_i_* | 1842.11 |
| NB^d^ | Negative-Binomial | **β**, *θ* | 2275.67 |
| NB Mixed^e^ | Negative-Binomial | **β**, *θ*, *v_i_* | 2275.70 |
| NB BYM^f^ | Negative-Binomial | **β**, *θ*, *v_i_, u_i_* | 1961.86 |

^a^Poisson Regression: $y_{i}|\lambda_{i}\sim\text{Poisson}(\lambda_{i}), \log(\lambda_{i})=\mathbf{\beta x}_{i}+\log(\mu_{i})$

^b^Poisson Mixed Effect Model: $y_{i}|\lambda_{i}\sim\text{Poisson}(\lambda_{i}), \log(\lambda_{i})=\mathbf{\beta x}_{i}+v_{i}+\log(\mu_{i})$

^c^Poisson Besag-York-Mollie Spatial Model: $y_{i}|\lambda_{i}\sim\text{Poisson}(\lambda_{i}), \log(\lambda_{i})=\mathbf{\beta x}_{i}+v_{i}+u_{i}+\log(\mu_{i})$

^d^Negative-Binomial Regression: $y_{i}|\lambda_{i}\sim\text{NBinomial}(\theta, \lambda_{i}), \log(\lambda_{i})=\mathbf{\beta x}_{i}+\log(\mu_{i})$

^e^Negative-Binomial Mixed Effect Model: $y_{i}|\lambda_{i}\sim\text{NBinomial}(\theta, \lambda_{i}), \log(\lambda_{i})=\mathbf{\beta x}_{i}+v_{i}+\log(\mu_{i})$

^f^Negative-Binomial Besag-York-Mollie Spatial Model: $y_{i}|\lambda_{i}\sim\text{NBinomial}(\theta, \lambda_{i}), \log(\lambda_{i})=\mathbf{\beta x}_{i}+v_{i}+u_{i}+\log(\mu_{i})$

^g^*y_i_* = observed death number in the *i*th municipality; *λ_i_* = expected death number in the *i*th municipality; *μ_i_* = population in the *i*th municipality (offset); **β** = regression coefficients for fixed effect; *v_i_* = region-specific random effect; *u_i_* = spatial random effect; *θ* = dispersion parameter.

^h^Deviance information criterion (DIC) measures the trade-off between model fit and model complexity.

**Table S3.** Hyperparameters and relevant descriptions for the two machine learning models implemented in this study.

| **Model^a^** | **Algorithm** | **Python Package^b^** | **Hyperparameter** |
| --- | --- | --- | --- |
| RF | Bagging | scikit-learn | n_estimators=600;  max_features=4;  min_samples_split=17 |
| XGBOOST | Boosting | xgboost | booster='gbtree';  n_estimators=100;  learning_rate=0.09;  max_depth=6;  min_child_weight=4.2;  gamma=0;  subsample=1;  colsample_bytree=0.82;  reg_lambda=1;  reg_alpha=5e-6 |

^a^RF = random forest; XGBOOST = extreme gradient boosting.

^b^scikit-learn: n_estimators is the number of trees in the forest; max_features is the number of features to consider when looking for the best split; min_samples_split is the minimum number of samples required to split an internal node.

xgboost: booster specifies which booster to use, e.g., tree-based model, linear model, etc; n_estimators is the number of gradient boosted trees; learning_rate is the boosting learning rate; max_depth is the maximum tree depth for base learners; min_child_weight is the minimum sum of instance weight needed in a child; gamma is the minimum loss reduction required to make a further partition on a leaf node of the tree; subsample is the subsample ratio of the training instance; colsample_bytree is the subsample ratio of columns when constructing each tree; reg_lambda is the L2 regularization term on weights; reg_alpha is the L1 regularization term on weights.

**Table S4.** Associations of municipality COVID-19 mortality rates with a range of socioexposomic factors, calculated from 8 geostatistical and machine learning models.

| **Variable Name** | **Poisson Regression** | **Poisson Mixed**  **Effect Model** | **Poisson BYM**  **Spatial Model** | **Negative-Binomial Regression** | **Negative-Binomial Mixed Effect Model** | **Negative-Binomial BYM Spatial Model** | **Random Forest** | **Gradient Boosting** | **Unit** |
| --- | --- | --- | --- | --- | --- | --- | --- | --- | --- |
| PM25 Average Conc. | 1.217 (1.149, 1.289) | 1.318 (1.21, 1.436) | 1.101 (0.882, 1.373) | 1.202 (0.972, 1.483) | 1.202 (0.971, 1.483) | 1.101 (0.884, 1.37) | 1.134 | 1.098 | 1 μg/m^3^ |
| NO2 Average Conc. | 1.033 (1.025, 1.04) | 1.037 (1.027, 1.045) | 1.064 (1.038, 1.092) | 1.054 (1.028, 1.082) | 1.054 (1.028, 1.082) | 1.064 (1.038, 1.092) | 1.048 | 1.044 | 1 ppb |
| Ozone Seasonal DM8HA | 0.875 (0.862, 0.889) | 0.854 (0.833, 0.875) | 0.908 (0.847, 0.971) | 0.879 (0.828, 0.932) | 0.879 (0.827, 0.932) | 0.908 (0.85, 0.969) | 0.907 | 0.87 | 1 ppb |
| Acrolein | 1.025 (1.014, 1.036) | 1.014 (1.001, 1.028) | 1.058 (1.004, 1.115) | 1.054 (0.999, 1.116) | 1.054 (0.999, 1.116) | 1.058 (1.005, 1.115) | 1.074 | 1.043 | 0.1 HQ |
| Acetaldehyde | 1.13 (1.099, 1.162) | 1.127 (1.093, 1.162) | 1.164 (1.048, 1.296) | 1.158 (1.045, 1.281) | 1.158 (1.045, 1.283) | 1.164 (1.048, 1.293) | 1.08 | 1.103 | 0.01 HQ |
| Formaldehyde | 1.115 (1.09, 1.141) | 1.119 (1.089, 1.149) | 1.153 (1.052, 1.264) | 1.168 (1.067, 1.276) | 1.168 (1.067, 1.278) | 1.153 (1.052, 1.262) | 1.108 | 1.082 | 0.01 HQ |
| Diesel PM | 1.021 (0.986, 1.059) | 0.964 (0.925, 1.005) | 1.103 (0.869, 1.37) | 1.121 (0.898, 1.43) | 1.12 (0.897, 1.429) | 1.126 (0.922, 1.376) | 1.542 | 1.589 | 0.1 HQ |
| Naphthalene | 1.486 (1.394, 1.584) | 1.478 (1.377, 1.582) | 1.669 (1.288, 2.166) | 1.74 (1.338, 2.266) | 1.74 (1.338, 2.268) | 1.672 (1.29, 2.168) | 1.529 | 1.728 | 0.01 HQ |
| Respiratory Hazard Index | 1.107 (1.081, 1.134) | 1.071 (1.04, 1.104) | 1.225 (1.088, 1.38) | 1.24 (1.091, 1.409) | 1.239 (1.091, 1.411) | 1.226 (1.09, 1.381) | 1.263 | 1.255 | 1 sd (0.08) |
| Inhalation Cancer Risk | 1.169 (1.13, 1.21) | 1.168 (1.123, 1.213) | 1.154 (1.01, 1.319) | 1.21 (1.065, 1.377) | 1.212 (1.065, 1.377) | 1.154 (1.01, 1.318) | 1.074 | 1.101 | 1 sd (4.2) |
| NPL Site Proximity | 1.074 (1.052, 1.094) | 1.066 (1.045, 1.089) | 1.117 (1.029, 1.213) | 1.096 (1.009, 1.191) | 1.096 (1.009, 1.191) | 1.117 (1.03, 1.213) | 1.057 | 1.069 | 20% quantile |
| RMP Facility Proximity | 1.042 (1.019, 1.065) | 1.048 (1.023, 1.073) | 1.069 (0.979, 1.169) | 1.075 (0.987, 1.171) | 1.075 (0.987, 1.171) | 1.069 (0.979, 1.168) | 1.03 | 1.005 | 20% quantile |
| TSDF Facility Proximity | 1.141 (1.114, 1.17) | 1.13 (1.1, 1.162) | 1.196 (1.096, 1.305) | 1.186 (1.089, 1.294) | 1.186 (1.088, 1.294) | 1.197 (1.097, 1.306) | 1.053 | 1.003 | 20% quantile |
| Proximity to TWWD | 1.076 (1.054, 1.096) | 1.077 (1.055, 1.099) | 1.102 (1.016, 1.194) | 1.124 (1.039, 1.217) | 1.124 (1.039, 1.217) | 1.102 (1.016, 1.194) | 1.026 | 1.043 | 20% quantile |
| Traffic Proximity | 1.1 (1.075, 1.125) | 1.09 (1.063, 1.116) | 1.153 (1.058, 1.256) | 1.092 (1.005, 1.189) | 1.092 (1.005, 1.188) | 1.153 (1.059, 1.256) | 1.089 | 1.074 | 20% quantile |
| Proximity to WFFEGU | 1.017 (1, 1.035) | 1.012 (0.991, 1.034) | 1 (0.918, 1.09) | 0.984 (0.901, 1.075) | 0.984 (0.9, 1.075) | 1 (0.919, 1.089) | 0.991 | 0.993 | 20% quantile |
| DOT Noise Level | 1.022 (1.002, 1.043) | 1.015 (0.994, 1.038) | 1.02 (0.898, 1.16) | 0.979 (0.864, 1.115) | 0.979 (0.863, 1.115) | 1.02 (0.898, 1.16) | 1.261 | 1.113 | 1 sd (2.7 dB) |
| Population Density | 1.065 (1.037, 1.094) | 1.044 (1.013, 1.077) | 1.172 (1.068, 1.287) | 1.1 (1.005, 1.204) | 1.1 (1.004, 1.204) | 1.171 (1.068, 1.285) | 1.082 | 1.068 | 20% quantile |
| % Population (Age < 15) | 1.105 (1.074, 1.138) | 1.095 (1.064, 1.129) | 1.082 (0.956, 1.226) | 1.017 (0.886, 1.169) | 1.017 (0.886, 1.169) | 1.083 (0.957, 1.226) | 1.011 | 1.024 | 1 sd (4.3%) |
| % Population (Age 15-44) | 0.675 (0.652, 0.698) | 0.688 (0.664, 0.712) | 0.724 (0.626, 0.839) | 0.685 (0.593, 0.795) | 0.685 (0.593, 0.795) | 0.725 (0.626, 0.839) | 0.868 | 0.705 | 1 sd (7.3%) |
| % Population (Age 45-64) | 1.037 (1.005, 1.068) | 1.069 (1.035, 1.105) | 1.033 (0.903, 1.181) | 1.09 (0.946, 1.252) | 1.091 (0.946, 1.254) | 1.033 (0.902, 1.181) | 0.956 | 0.946 | 1 sd (4.2%) |
| % Population (Age > 64) | 1.582 (1.537, 1.629) | 1.554 (1.504, 1.605) | 1.438 (1.246, 1.652) | 1.522 (1.322, 1.756) | 1.522 (1.322, 1.756) | 1.438 (1.246, 1.652) | 1.313 | 1.749 | 1 sd (7.9%) |
| % Population (White) | 0.894 (0.869, 0.919) | 0.878 (0.852, 0.905) | 0.814 (0.713, 0.926) | 0.871 (0.762, 0.993) | 0.871 (0.762, 0.993) | 0.813 (0.713, 0.926) | 0.649 | 0.666 | 1 sd (19%) |
| % Population (Black) | 1.07 (1.043, 1.099) | 1.073 (1.044, 1.101) | 1.087 (0.959, 1.231) | 1.024 (0.908, 1.162) | 1.024 (0.907, 1.162) | 1.088 (0.959, 1.232) | 1.072 | 1.138 | 1 sd (13.6%) |
| % Population (Asian) | 1.016 (0.994, 1.038) | 1.03 (1.008, 1.054) | 1.134 (1.012, 1.271) | 1.182 (1.048, 1.34) | 1.182 (1.048, 1.34) | 1.134 (1.012, 1.273) | 1.298 | 1.201 | 1 sd (7.8%) |
| % Population (Hispanic) | 1.103 (1.083, 1.123) | 1.106 (1.085, 1.126) | 1.244 (1.11, 1.397) | 1.146 (1.024, 1.289) | 1.146 (1.024, 1.289) | 1.245 (1.11, 1.398) | 1.31 | 1.228 | 1 sd (13.7%) |
| % Minority | 1.234 (1.197, 1.273) | 1.276 (1.235, 1.32) | 1.422 (1.24, 1.634) | 1.271 (1.107, 1.462) | 1.271 (1.107, 1.462) | 1.423 (1.24, 1.636) | 1.464 | 1.415 | 1 sd (23.4%) |
| % Below High School Edu. | 1.162 (1.137, 1.188) | 1.166 (1.14, 1.194) | 1.363 (1.201, 1.548) | 1.262 (1.112, 1.438) | 1.262 (1.112, 1.438) | 1.363 (1.201, 1.55) | 1.277 | 1.397 | 1 sd (6.3%) |
| % Linguistic Isolate | 1.155 (1.135, 1.176) | 1.162 (1.141, 1.182) | 1.301 (1.163, 1.455) | 1.206 (1.074, 1.361) | 1.206 (1.073, 1.361) | 1.301 (1.163, 1.456) | 1.096 | 1.103 | 1 sd (5%) |
| % Below Poverty Level | 1.091 (1.053, 1.13) | 1.065 (1.025, 1.105) | 1.119 (0.951, 1.314) | 1.041 (0.881, 1.23) | 1.041 (0.881, 1.23) | 1.117 (0.951, 1.314) | 1.008 | 1.02 | 1 sd (12.1%) |
| Gini Index | 1.051 (1.017, 1.087) | 1.057 (1.021, 1.094) | 1.026 (0.904, 1.165) | 1.065 (0.935, 1.213) | 1.065 (0.935, 1.213) | 1.026 (0.904, 1.164) | 1.115 | 1.088 | 1 sd (0.05) |
| Median Gross Rent | 1.074 (1.038, 1.111) | 1.088 (1.05, 1.127) | 1.234 (1.09, 1.398) | 1.2 (1.063, 1.359) | 1.2 (1.063, 1.359) | 1.235 (1.09, 1.398) | 1.063 | 1.099 | 1 sd ($347) |
| Median Household Income | 0.985 (0.964, 1.006) | 1.005 (0.982, 1.029) | 1.014 (0.917, 1.123) | 1.041 (0.944, 1.147) | 1.041 (0.944, 1.147) | 1.014 (0.918, 1.122) | 1 | 0.998 | 20% quantile |
| Median House Value | 0.912 (0.872, 0.954) | 0.931 (0.889, 0.972) | 0.849 (0.727, 0.988) | 0.827 (0.705, 0.969) | 0.827 (0.705, 0.97) | 0.85 (0.728, 0.989) | 1.199 | 1.167 | 1 sd ($20w) |
| % High Occupancy Residence | 1.176 (1.147, 1.204) | 1.16 (1.131, 1.188) | 1.09 (0.966, 1.23) | 1.273 (1.122, 1.455) | 1.273 (1.122, 1.455) | 1.091 (0.967, 1.23) | 1.208 | 1.304 | 1 sd (8.6%) |
| % Group Quarter Residence | 1.053 (1.018, 1.089) | 1.075 (1.039, 1.111) | 1.042 (0.927, 1.168) | 1.328 (1.138, 1.571) | 1.328 (1.138, 1.573) | 1.042 (0.927, 1.168) | 1.25 | 1.235 | 1 sd (6.5%) |
| % Population (Disability) | 1.007 (0.963, 1.052) | 0.965 (0.919, 1.012) | 1.17 (1, 1.372) | 1.146 (0.982, 1.339) | 1.146 (0.982, 1.339) | 1.17 (1, 1.372) | 0.986 | 0.985 | 1 sd (4.2%) |
| % Uninsured | 1.177 (1.151, 1.203) | 1.179 (1.153, 1.206) | 1.23 (1.085, 1.394) | 1.12 (0.989, 1.274) | 1.12 (0.989, 1.273) | 1.23 (1.087, 1.394) | 1.087 | 1.071 | 1 sd (4.4%) |
| % Unemployed | 0.895 (0.869, 0.921) | 0.885 (0.858, 0.912) | 0.844 (0.744, 0.957) | 0.863 (0.759, 0.982) | 0.863 (0.759, 0.982) | 0.844 (0.744, 0.957) | 0.985 | 1.028 | 1 sd (2.6%) |
| % Occupation (High Risk) | 1.052 (1.022, 1.084) | 1.062 (1.03, 1.094) | 1.075 (0.953, 1.212) | 1.139 (1.007, 1.288) | 1.139 (1.007, 1.288) | 1.075 (0.954, 1.21) | 0.957 | 0.962 | 1 sd (3.9%) |
| % Commute (To Diff. Counties) | 0.943 (0.906, 0.98) | 0.911 (0.873, 0.95) | 0.983 (0.836, 1.155) | 1.022 (0.868, 1.201) | 1.022 (0.868, 1.202) | 0.984 (0.837, 1.155) | 1 | 1.011 | 1 sd (7.7%) |
| % Commute (To NY City) | 0.98 (0.959, 1.001) | 0.968 (0.946, 0.99) | 1.025 (0.899, 1.168) | 1.049 (0.908, 1.231) | 1.049 (0.908, 1.231) | 1.025 (0.9, 1.168) | 1.42 | 1.539 | 1 sd (3.8%) |
| % Commute (Public Transport) | 1.002 (0.981, 1.022) | 0.988 (0.967, 1.011) | 0.982 (0.866, 1.113) | 1.014 (0.887, 1.17) | 1.014 (0.887, 1.17) | 0.982 (0.866, 1.113) | 1.193 | 1.121 | 1 sd (4.5%) |
| SVI (Overall) | 1.435 (1.381, 1.49) | 1.501 (1.438, 1.567) | 1.711 (1.489, 1.97) | 1.657 (1.443, 1.904) | 1.657 (1.443, 1.906) | 1.711 (1.489, 1.97) | 1.296 | 1.303 | 1 sd (0.29) |

**Table S5.** Comparison of association estimates from the 6 geostatistical models developed in the Frequentist framework vs the Bayesian framework.

| **Model** | **Poisson Regression** | | **Poisson Mixed**  **Effect Model** | | **Poisson BYM**  **Spatial Model** | | **Negative-Binomial Regression** | | **Negative-Binomial**  **Mixed Effect Model** | | **Negative-Binomial BYM Spatial Model** | |
| --- | --- | --- | --- | --- | --- | --- | --- | --- | --- | --- | --- | --- |
|  | Bayesian | Frequentist | Bayesian | Frequentist | Bayesian | Frequentist | Bayesian | Frequentist | Bayesian | Frequentist | Bayesian | Frequentist |
|  | INLA (R) | stats (R) | INLA (R) | lme4 (R) | INLA (R) | spaMM (R) | INLA (R) | stats (R) | INLA (R) | lme4 (R) | INLA (R) | spaMM (R) |
| % Population (Age > 64) | 1.58  (1.53, 1.63) | 1.58  (1.53, 1.63) | 1.55  (1.50, 1.60) | 1.54  (1.50, 1.60) | 1.42  (1.23, 1.64) | 1.43  (1.25, 1.65) | 1.51  (1.33, 1.75) | 1.52  (1.33, 1.73) | 1.51  (1.32, 1.75) | 1.52  (1.32, 1.74) | 1.42  (1.23, 1.64) | 1.43  (1.25, 1.65) |
| % Minority | 1.23  (1.20, 1.27) | 1.23  (1.20, 1.27) | 1.28  (1.24, 1.32) | 1.28  (1.24, 1.32) | 1.42  (1.24, 1.63) | 1.42  (1.25, 1.62) | 1.27  (1.11, 1.46) | 1.27  (1.11, 1.46) | 1.27  (1.11, 1.46) | 1.27  (1.11, 1.46) | 1.42  (1.24, 1.64) | 1.41  (1.23, 1.61) |
| % Below High School Edu. | 1.16  (1.14, 1.19) | 1.16  (1.14, 1.19) | 1.17  (1.14, 1.19) | 1.17  (1.14, 1.19) | 1.36  (1.20, 1.55) | 1.36  (1.22, 1.54) | 1.26  (1.11, 1.44) | 1.26  (1.11, 1.43) | 1.26  (1.11, 1.44) | 1.26  (1.11, 1.43) | 1.36  (1.20, 1.55) | 1.36  (1.21, 1.54) |
| Median Gross Rent | 1.07  (1.04, 1.11) | 1.07  (1.04, 1.11) | 1.09  (1.05, 1.13) | 1.09  (1.05, 1.13) | 1.23  (1.09, 1.40) | 1.24  (1.10, 1.40) | 1.20  (1.06, 1.36) | 1.20  (1.07, 1.35) | 1.20  (1.06, 1.36) | 1.20  (1.06, 1.36) | 1.23  (1.09, 1.40) | 1.24  (1.10, 1.40) |
| Population Density | 1.07  (1.04, 1.09) | 1.07  (1.04, 1.09) | 1.04  (1.01, 1.08) | 1.04  (1.01, 1.08) | 1.17  (1.07, 1.29) | 1.18  (1.08, 1.29) | 1.10  (1,01, 1.20) | 1.09  (1.01, 1.19) | 1.10  (1.00, 1.20) | 1.09  (1.00, 1.19) | 1.17  (1.07, 1.29) | 1.18  (1.08, 1.29) |
| % Occupation (High Risk) | 1.05  (1.02, 1.08) | 1.05  (1.02, 1.08) | 1.06  (1.03, 1.09) | 1.06  (1.03, 1.09) | 1.07  (0.95, 1.21) | 1.08  (0.96, 1.21) | 1.14  (1.01, 1.29) | 1.14  (1.02, 1.28) | 1.14  (1.01, 1.29) | 1.14  (1.01, 1.29) | 1.07  (0.95, 1.21) | 1.08  (0.96, 1.21) |
| PM25 Average Conc. | 1.22  (1.15, 1.29) | 1.22  (1.15, 1.29) | 1.33  (1.22, 1.46) | 1.34  (1.22, 1.46) | 1.09  (0.87, 1.37) | 1.08  (0.87, 1.34) | 1.20  (0.96, 1.49) | 1.20  (0.96, 1.49) | 1.20  (0.96, 1.49) | 1.20  (0.97, 1.49) | 1.09  (0.87, 1.36) | 1.08  (0.87, 1.34) |
| Ozone Seasonal DM8HA | 0.88  (0.86, 0.89) | 0.88  (0.86, 0.89) | 0.85  (0.83, 0.88) | 0.85  (0.83, 0.88) | 0.91  (0.85, 0.97) | 0.91  (0.86, 0.97) | 0.88  (0.83, 0.93) | 0.88  (0.83, 0.94) | 0.88  (0.83, 0.93) | 0.88  (0.83, 0.93) | 0.91  (0.85, 0.97) | 0.91  (0.86, 0.97) |
| % High Occupancy Residence | 1.17  (1.15, 1.20) | 1.17  (1.15, 1.20) | 1.16  (1.13, 1.19) | 1.16  (1.13, 1.19) | 1.09  (0.96, 1.22) | 1.09  (0.97, 1.23) | 1.27  (1.12, 1.45) | 1.26  (1.13, 1.42) | 1.27  (1.12, 1.45) | 1.26  (1.11, 1.44) | 1.09  (0.96, 1.23) | 1.09  (0.97, 1.23) |
| % Unemployed | 0.90  (0.87, 0.92) | 0.90  (0.87, 0.92) | 0.88  (0.86, 0.91) | 0.88  (0.86, 0.91) | 0.84  (0.74, 0.95) | 0.84  (0.74, 0.95) | 0.86  (0.76, 0.98) | 0.86  (0.76, 0.97) | 0.86  (0.76, 0.98) | 0.86  (0.76, 0.98) | 0.84  (0.74, 0.95) | 0.84  (0.74, 0.95) |

**Table S6.** Computational time for fitting/training single models with different algorithms.

| **Computational Framework** | **Statistical Learning** | | | | | | | | | | | | **Machine Learning** | |
| --- | --- | --- | --- | --- | --- | --- | --- | --- | --- | --- | --- | --- | --- | --- |
| **Model** | **Poisson Regression** | | **Negative-Binomial Regression** | | **Poisson Mixed**  **Effect Model** | | **Negative-Binomial Mixed Effect Model** | | **Poisson BYM**  **Spatial Model** | | **Negative-Binomial BYM Spatial Model** | | **Random Forest** | **Gradient Boosting** |
| **Estimation** | Bayesian | Frequentist | Bayesian | Frequentist | Bayesian | Frequentist | Bayesian | Frequentist | Bayesian | Frequentist | Bayesian | Frequentist | Bagging | Boosting |
| **Package** | INLA (R) | stats (R) | INLA (R) | stats (R) | INLA (R) | lme4 (R) | INLA (R) | lme4 (R) | INLA (R) | spaMM (R) | INLA (R) | spaMM (R) | scikit-learn (Python) | xgboost (Python) |
| **Computational Time (sec.)** | 3.71±0.42 | 0.02±0.01 | 3.86±0.23 | 0.09±0.01 | 4.10±0.29 | 1.21±0.02 | 5.11±0.10 | 49.94±0.34 | 11.04±0.35 | 23.49±0.39 | 9.02±0.53 | 60.63±2.50 | 1.02±0.02 | 0.16±0.01 |

**Table S7.** True regression coefficients and the corresponding estimates from 8 statistical and geospatial models for Scenario 1 $(r_{1}=0.6, r_{2}=0.6)$.

|  | **True regression coefficient** | **Poisson Regression**  **(565 samples)** | **Poisson Mixed**  **Effect Model**  **(565 samples)** | **Poisson BYM**  **Spatial Model**  **(565 samples)** | **Poisson BYM**  **Spatial Model**  **(356 samples)** | **Negative-Binomial Regression**  **(565 samples)** | **Negative-Binomial Mixed Effect Model**  **(565 samples)** | **Negative-Binomial BYM Spatial Model (565 samples)** | **Negative-Binomial BYM Spatial Model (356 samples)** |
| --- | --- | --- | --- | --- | --- | --- | --- | --- | --- |
| Intercept | -5 | -2.35 (-2.7, -2) | -9.29 (-9.95, -8.63) | -4.11 (-5.78, -2.44) | -4.83 (-6.87, -2.8) | -3.92 (-5.77, -2.07) | -3.92 (-5.77, -2.07) | -4.09 (-5.8, -2.39) | -4.81 (-6.93, -2.69) |
| *X*_1_ | 0.5 | 0.45 (0.43, 0.47) | 0.47 (0.45, 0.49) | 0.47 (0.38, 0.56) | 0.52 (0.38, 0.65) | 0.49 (0.41, 0.58) | 0.49 (0.41, 0.58) | 0.47 (0.38, 0.56) | 0.52 (0.38, 0.66) |
| *X*_2_ | 0.35 | 0.42 (0.4, 0.43) | 0.46 (0.44, 0.47) | 0.38 (0.28, 0.47) | 0.48 (0.37, 0.6) | 0.48 (0.38, 0.58) | 0.48 (0.38, 0.58) | 0.38 (0.28, 0.47) | 0.48 (0.36, 0.6) |
| *X*_3_ | 0.2 | 0.09 (0.08, 0.11) | 0.09 (0.08, 0.1) | 0.12 (0.02, 0.22) | 0.06 (-0.05, 0.18) | 0.07 (-0.03, 0.17) | 0.07 (-0.03, 0.17) | 0.12 (0.02, 0.22) | 0.06 (-0.06, 0.18) |
| *X*_4_ | 0.2 | 0.12 (0.11, 0.13) | 0.1 (0.09, 0.12) | 0.16 (0.08, 0.23) | 0.12 (0.01, 0.23) | 0.14 (0.06, 0.21) | 0.14 (0.06, 0.21) | 0.16 (0.08, 0.23) | 0.12 (0.01, 0.24) |
| *X*_5_ | 0.25 | 0.01 (-0.01, 0.02) | 0.08 (0.06, 0.09) | 0.21 (0.14, 0.29) | 0.24 (0.14, 0.34) | 0.16 (0.08, 0.23) | 0.16 (0.08, 0.23) | 0.22 (0.14, 0.29) | 0.24 (0.13, 0.34) |
| *X*_6_ | 0.05 | -0.14 (-0.16, -0.12) | -0.15 (-0.17, -0.13) | 0.05 (-0.04, 0.14) | 0.02 (-0.14, 0.18) | 0.03 (-0.06, 0.13) | 0.03 (-0.06, 0.13) | 0.05 (-0.04, 0.15) | 0.02 (-0.14, 0.18) |
| *X*_7_ | 0.15 | 0.3 (0.27, 0.32) | 0.63 (0.6, 0.66) | 0.15 (0.02, 0.27) | 0.14 (-0.03, 0.31) | 0.17 (0.04, 0.3) | 0.17 (0.04, 0.3) | 0.14 (0.02, 0.27) | 0.14 (-0.03, 0.32) |
| *X*_8_ | -0.05 | -0.13 (-0.14, -0.13) | -0.03 (-0.05, -0.02) | -0.07 (-0.11, -0.03) | -0.05 (-0.1, -0.01) | -0.07 (-0.12, -0.03) | -0.07 (-0.12, -0.03) | -0.07 (-0.11, -0.03) | -0.06 (-0.1, -0.01) |
| *X*_9_ | 0.15 | 0.12 (0.11, 0.13) | 0.12 (0.11, 0.13) | 0.17 (0.1, 0.25) | 0.17 (0.08, 0.26) | 0.14 (0.07, 0.22) | 0.14 (0.07, 0.22) | 0.17 (0.1, 0.25) | 0.17 (0.08, 0.27) |
| *X*_10_ | -0.5 | -0.24 (-0.25, -0.23) | -0.25 (-0.26, -0.24) | -0.52 (-0.59, -0.44) | -0.54 (-0.64, -0.45) | -0.52 (-0.6, -0.44) | -0.52 (-0.6, -0.44) | -0.52 (-0.6, -0.44) | -0.54 (-0.64, -0.44) |

**Table S8.** True regression coefficients and the corresponding estimates from 8 statistical and geospatial models for Scenario 2 $(r_{1}=0.6, r_{2}=0.3)$.

|  | **True regression coefficient** | **Poisson Regression**  **(565 samples)** | **Poisson Mixed**  **Effect Model**  **(565 samples)** | **Poisson BYM**  **Spatial Model**  **(565 samples)** | **Poisson BYM**  **Spatial Model**  **(356 samples)** | **Negative-Binomial Regression**  **(565 samples)** | **Negative-Binomial Mixed Effect Model**  **(565 samples)** | **Negative-Binomial BYM Spatial Model (565 samples)** | **Negative-Binomial BYM Spatial Model (356 samples)** |
| --- | --- | --- | --- | --- | --- | --- | --- | --- | --- |
| Intercept | -5 | -0.61 (-0.96, -0.26) | -4.22 (-4.8, -3.63) | -4.54 (-6.33, -2.76) | -5.48 (-7.61, -3.35) | -3.98 (-5.76, -2.19) | -3.98 (-5.76, -2.19) | -4.49 (-6.3, -2.68) | -5.48 (-7.72, -3.24) |
| *X*_1_ | 0.5 | 0.43 (0.41, 0.45) | 0.42 (0.4, 0.43) | 0.55 (0.47, 0.63) | 0.55 (0.42, 0.68) | 0.56 (0.48, 0.64) | 0.56 (0.48, 0.64) | 0.55 (0.47, 0.64) | 0.55 (0.41, 0.69) |
| *X*_2_ | 0.35 | 0.24 (0.22, 0.25) | 0.25 (0.23, 0.26) | 0.37 (0.27, 0.46) | 0.46 (0.34, 0.58) | 0.42 (0.33, 0.52) | 0.42 (0.33, 0.52) | 0.37 (0.27, 0.47) | 0.46 (0.34, 0.59) |
| *X*_3_ | 0.2 | 0.14 (0.13, 0.15) | 0.14 (0.13, 0.15) | 0.14 (0.04, 0.23) | 0.08 (-0.03, 0.2) | 0.1 (0.01, 0.2) | 0.1 (0.01, 0.2) | 0.14 (0.04, 0.24) | 0.08 (-0.04, 0.2) |
| *X*_4_ | 0.2 | 0.03 (0.01, 0.04) | 0.02 (0, 0.03) | 0.13 (0.05, 0.2) | 0.11 (0, 0.22) | 0.09 (0.02, 0.16) | 0.09 (0.02, 0.16) | 0.12 (0.05, 0.2) | 0.11 (-0.01, 0.22) |
| *X*_5_ | 0.25 | 0.09 (0.08, 0.11) | 0.07 (0.05, 0.09) | 0.21 (0.13, 0.28) | 0.21 (0.11, 0.31) | 0.17 (0.09, 0.24) | 0.17 (0.09, 0.24) | 0.21 (0.13, 0.29) | 0.21 (0.1, 0.31) |
| *X*_6_ | 0.05 | -0.15 (-0.18, -0.13) | -0.14 (-0.17, -0.12) | 0.04 (-0.05, 0.13) | 0.04 (-0.12, 0.2) | 0.03 (-0.06, 0.11) | 0.03 (-0.06, 0.11) | 0.04 (-0.05, 0.13) | 0.04 (-0.13, 0.2) |
| *X*_7_ | 0.15 | 0.34 (0.32, 0.37) | 0.54 (0.51, 0.57) | 0.18 (0.05, 0.31) | 0.21 (0.04, 0.38) | 0.18 (0.06, 0.31) | 0.18 (0.06, 0.31) | 0.18 (0.05, 0.31) | 0.21 (0.03, 0.39) |
| *X*_8_ | -0.05 | -0.18 (-0.19, -0.18) | -0.14 (-0.15, -0.12) | -0.07 (-0.11, -0.03) | -0.05 (-0.1, 0) | -0.08 (-0.12, -0.04) | -0.08 (-0.12, -0.04) | -0.07 (-0.11, -0.03) | -0.05 (-0.1, 0) |
| *X*_9_ | 0.15 | 0.16 (0.15, 0.17) | 0.16 (0.15, 0.17) | 0.16 (0.09, 0.24) | 0.16 (0.07, 0.25) | 0.15 (0.08, 0.22) | 0.15 (0.08, 0.22) | 0.16 (0.09, 0.24) | 0.16 (0.06, 0.25) |
| *X*_10_ | -0.5 | -0.24 (-0.25, -0.23) | -0.24 (-0.26, -0.23) | -0.55 (-0.63, -0.48) | -0.56 (-0.66, -0.47) | -0.55 (-0.62, -0.47) | -0.55 (-0.62, -0.47) | -0.56 (-0.64, -0.48) | -0.56 (-0.66, -0.46) |

**Table S9.** True regression coefficients and the corresponding estimates from 8 statistical and geospatial models for Scenario 3 $(r_{1}=0.3, r_{2}=0.6)$.

|  | **True regression coefficient** | **Poisson Regression**  **(565 samples)** | **Poisson Mixed**  **Effect Model**  **(565 samples)** | **Poisson BYM**  **Spatial Model**  **(565 samples)** | **Poisson BYM**  **Spatial Model**  **(356 samples)** | **Negative-Binomial Regression**  **(565 samples)** | **Negative-Binomial Mixed Effect Model**  **(565 samples)** | **Negative-Binomial BYM Spatial Model (565 samples)** | **Negative-Binomial BYM Spatial Model (356 samples)** |
| --- | --- | --- | --- | --- | --- | --- | --- | --- | --- |
| Intercept | -5 | -2.9 (-3.12, -2.69) | -13.69 (-14.55, -12.84) | -4.1 (-6.9, -1.31) | -2.25 (-5.5, 1) | -4.78 (-7.85, -1.71) | -4.78 (-7.85, -1.7) | -4.11 (-6.98, -1.24) | -4.49 (-8.03, -0.95) |
| *X*_1_ | 0.5 | 0.8 (0.79, 0.81) | 0.79 (0.78, 0.8) | 0.55 (0.41, 0.68) | 0.88 (0.68, 1.09) | 0.69 (0.56, 0.81) | 0.69 (0.56, 0.81) | 0.55 (0.41, 0.69) | 0.57 (0.34, 0.79) |
| *X*_2_ | 0.35 | 0.81 (0.8, 0.82) | 0.86 (0.85, 0.87) | 0.38 (0.22, 0.55) | 0.95 (0.76, 1.14) | 0.78 (0.61, 0.95) | 0.78 (0.61, 0.95) | 0.38 (0.21, 0.56) | 0.52 (0.32, 0.73) |
| *X*_3_ | 0.2 | -0.09 (-0.1, -0.08) | -0.08 (-0.09, -0.08) | 0.1 (-0.07, 0.27) | -0.11 (-0.3, 0.08) | -0.08 (-0.25, 0.08) | -0.08 (-0.25, 0.08) | 0.1 (-0.07, 0.27) | 0.03 (-0.18, 0.24) |
| *X*_4_ | 0.2 | -0.03 (-0.04, -0.02) | -0.16 (-0.18, -0.15) | 0.1 (-0.03, 0.22) | -0.04 (-0.22, 0.14) | -0.08 (-0.2, 0.05) | -0.08 (-0.2, 0.05) | 0.1 (-0.03, 0.23) | 0.13 (-0.06, 0.33) |
| *X*_5_ | 0.25 | -0.46 (-0.47, -0.45) | -0.21 (-0.22, -0.2) | 0.19 (0.07, 0.32) | -0.61 (-0.77, -0.45) | -0.07 (-0.2, 0.06) | -0.07 (-0.2, 0.06) | 0.2 (0.07, 0.33) | 0.22 (0.04, 0.39) |
| *X*_6_ | 0.05 | -0.55 (-0.57, -0.54) | -0.66 (-0.68, -0.65) | 0.05 (-0.07, 0.17) | -0.61 (-0.86, -0.36) | -0.06 (-0.18, 0.07) | -0.06 (-0.18, 0.07) | 0.05 (-0.07, 0.18) | 0.1 (-0.17, 0.37) |
| *X*_7_ | 0.15 | 0.7 (0.68, 0.71) | 0.78 (0.76, 0.81) | 0.13 (-0.07, 0.34) | 0.72 (0.45, 0.99) | 0.28 (0.06, 0.5) | 0.28 (0.06, 0.5) | 0.13 (-0.08, 0.34) | 0.17 (-0.13, 0.46) |
| *X*_8_ | -0.05 | -0.18 (-0.19, -0.18) | 0.05 (0.04, 0.06) | -0.07 (-0.13, -0.01) | -0.21 (-0.28, -0.13) | -0.06 (-0.13, 0.01) | -0.06 (-0.13, 0.01) | -0.07 (-0.13, 0) | -0.07 (-0.15, 0.02) |
| *X*_9_ | 0.15 | 0.11 (0.1, 0.11) | 0.11 (0.1, 0.11) | 0.2 (0.07, 0.33) | 0.11 (-0.05, 0.26) | 0.15 (0.03, 0.27) | 0.15 (0.03, 0.27) | 0.2 (0.07, 0.34) | 0.17 (0.01, 0.34) |
| *X*_10_ | -0.5 | 0.06 (0.05, 0.07) | 0.09 (0.08, 0.1) | -0.55 (-0.68, -0.43) | 0.04 (-0.1, 0.19) | -0.52 (-0.64, -0.39) | -0.52 (-0.64, -0.39) | -0.56 (-0.69, -0.43) | -0.58 (-0.74, -0.42) |

**REFERENCES**

1. Di Q, Amini H, Shi L, Kloog I, Silvern R, Kelly J, et al. Assessing NO2 concentration and model uncertainty with high spatiotemporal resolution across the contiguous United States using ensemble model averaging. Environ Sci Technol. 2019;54:1372−1384.

2. Petroni M, Hill D, Younes L, Barkman L, Howard S, Howell IB, et al. Hazardous air pollutant exposure as a contributing factor to COVID-19 mortality in the United States. Environ Res Lett. 2020;15:0940a0949.

3. Agier L, Portengen L, Chadeau-Hyam M, Basagaña X, Giorgis-Allemand L, Siroux V, et al. A systematic comparison of linear regression–based statistical methods to assess exposome-health associations. Environ Health Perspect. 2016;124:1848–1856.

4. Barrera-Gómez J, Agier L, Portengen L, Chadeau-Hyam M, Giorgis-Allemand L, Siroux V, et al. A systematic comparison of statistical methods to detect interactions in exposome-health associations. Environ Health. 2017;16:1–13.

5. Sun Z, Tao Y, Li S, Ferguson KK, Meeker JD, Park SK, et al. Statistical strategies for constructing health risk models with multiple pollutants and their interactions: possible choices and comparisons. Environ Health. 2013;12:1–19.

1. ^*^Corresponding author at: Environmental and Occupational Health Sciences Institute (EOHSI), Rutgers University, Piscataway, NJ 08854, USA.

   Email: [panosg@ccl.rutgers.edu](mailto:panosg@ccl.rutgers.edu) (P.G. Georgopoulos). [↑](#footnote-ref-2)
